# Precision modeling of gall bladder cancer patients in mice based on orthotopic implantation of organoid-derived tumor buds

**DOI:** 10.1038/s41389-021-00322-1

**Published:** 2021-04-17

**Authors:** Shingo Kato, Kentaro Fushimi, Yuichiro Yabuki, Yoshiaki Maru, Sho Hasegawa, Tetsuya Matsuura, Daisuke Kurotaki, Akihiro Suzuki, Noritoshi Kobayashi, Masato Yoneda, Takuma Higurashi, Makiko Enaka, Tomohiko Tamura, Yoshitaka Hippo, Atsushi Nakajima

**Affiliations:** 1 Department of Clinical Cancer Genomics, Yokohama City University Hospital, Kanagawa, 236-0004 Japan; 2 Department of Gastroenterology and Hepatology, Yokohama City University Graduate School of Medicine, Kanagawa, 236-0004 Japan; 3 Department of Immunology, Yokohama City University Graduate School of Medicine, Kanagawa, 236-0004 Japan; 4 Department of Plastic and Reconstructive Surgery, Yokohama City University Graduate School of Medicine, Kanagawa, 236-0004 Japan; 5 Department of Molecular Carcinogenesis, Chiba Cancer Center Research Institute, Chiba, 260-8717 Japan; 6 Department of Oncology, Yokohama City University Hospital, Kanagawa, 236-0004 Japan; 7 Department of Molecular Pathology, Yokohama City University Graduate School of Medicine, Kanagawa, 236-0004 Japan

**Keywords:** Cancer models, Biliary tract cancer

## Abstract

Genetically engineered mice (GEM) are the gold standard for cancer modeling. However, strict recapitulation of stepwise carcinogenesis from a single tumor-initiating epithelial cell among genetically intact cells in adults is not feasible with the currently available techniques using GEM. In previous studies, we partially overcame this challenge by physically isolating organs from adult animals, followed by genetic engineering in organoids and subcutaneous inoculation in nude mice. Despite the establishment of suitable ex vivo carcinogenesis models for diverse tissues, tumor development remained ectopic and occurred under immunodeficient conditions. Further refinement was, therefore, necessary to establish ideal models. Given the poor prognosis and few models owing to the lack of gall bladder (GB)-specific *Cre* strain, we assumed that the development of authentic models would considerably benefit GB cancer research. Here, we established a novel model using GB organoids with mutant *Kras* and *Trp53* loss generated in vitro by lentiviral *Cre* transduction and CRISPR/Cas9 gene editing, respectively. Organoid-derived subcutaneous tumor fragments were sutured to the outer surface of the GB in syngeneic mice, which developed orthotopic tumors that resembled human GB cancer in histological and transcriptional features. This model revealed the infiltration of similar subsets of immune cells in both subcutaneous and orthotopic tumors, confirming the appropriate immune environment during carcinogenesis. In addition, we accurately validated the in vivo efficacy of gemcitabine, a common therapeutic agent for GB cancer, in large cohorts. Taken together, this model may serve as a promising avatar of patients with GB cancer in drug discovery and precision medicine.

## Introduction

Carcinogenesis is primarily driven by accumulated genetic alterations in epithelial cells but is also influenced by mutual interactions among non-malignant epithelial cells, stromal cells, and immune cells in the tissue microenvironment. As an experimental system integrating both genetic factors and tissue-specific microenvironment, genetically engineered mice (GEM) are an excellent modality for recapitulating multi-step carcinogenesis in a physiological setting. The Cre-loxP technology can further enable organ- or cell lineage-specific and inducible gene recombination, expanding its application to the development of spontaneous carcinogenesis models in space- or time-regulated manner^1^. GEM studies have long provided vital insights into the mechanisms underlying carcinogenesis, thereby accelerating research into cancer prevention and therapy. Despite these advantages, there are drawbacks to the use of GEM. First, the generation of mice models is usually time consuming and labor-intensive, especially in cases where multiple tissue-specific genetic aberrations are required for tumorigenesis. Second, tissue- or cell lineage-specific genetic engineering in floxed conditional alleles may not always be achieved with high accuracy. Although *Cre* expression is usually driven by the promoters of genes that are expressed in a limited number of tissue types or cell lineages, highly specific markers are yet to be identified in many organs. Third, only a subset of cells develops tumors amid seemingly normal cells that have already undergone gene recombination. However, this is not the case in the pathogenesis of sporadic cancer, wherein a single cell eventually develops a full-blown tumor among genetically intact epithelial cells.

To provide an alternative modality for disease modeling, we previously developed an ex vivo carcinogenesis model based on an organoid culture technique. The model comprises three units: an efficient in vitro gene lentivirus-mediated transduction^2^ based on the matrigel bilayer organoid culture protocol^3^, reconstitution of cancer-specific genetic aberrations in normal organoids from corresponding murine organs, and subcutaneous tumor development by the inoculation of organoids in immunodeficient mice. Such models have been established for the intestine^4^, lungs^5^, biliary tract^6^, and pancreas^7^, in which mutations in either *APC* or *KRAS* are predominant in human cancer patients. We demonstrated that organoid-based models could essentially recapitulate the results obtained in earlier studies with GEM when the incidence, size, and histological findings of tumors were measured as indicators of tumorigenicity^3^. More recently, we showed that chemical carcinogenesis could also be integrated into this model^8^. Based on these findings, we concluded that this approach may be a useful tool in cancer research as an alternative or complementary method to conventional GEM-based approaches.

However, this approach has certain limitations regarding the non-physiological conditions during tumor development. Thus, we postulated that carcinogenesis modeling should be conducted (a) from a single-cell epithelial cell through stepwise genetic alterations, (b) in the vicinity of or within the same organ that mostly comprises wild type (WT) cells, and (c) in the context of an intact immune system in syngeneic host mice. As a target organ, the gall bladder (GB) could provide a tissue-specific carcinogenesis model with the greatest benefit because GB cancer is an aggressive malignancy of the biliary tract with a refractory nature and poor prognosis, with few disease models with low tissue specificity established so far^9,10^. Among recurrently mutated genes in human GB cancer, *TP53* is the most frequently mutated gene, followed by *SMAD4*, *ARID1A*, *PIK3CA*, and *KRAS*^11–13^, whereas *ERBB2* amplification is also prevalent^14^. Among these mutations, we previously demonstrated that lentivirally induced *Kras*^*G12D*^ expression and p53 loss in GB organoids led to the development of adenocarcinoma-like solid subcutaneous tumors in nude mice with complete penetrance^6^.

In this study, we extended the organoid-based approach to develop a novel model for GB cancer-bearing patients. Owing to its highly scalable nature in immunologically and genetically intact conditions, this model will likely accelerate basic and translational studies on GB cancer.

## Results

### In vitro generation of p53-inactivated GB organoids expressing oncogenic *Kras*

**Fig. 1:**
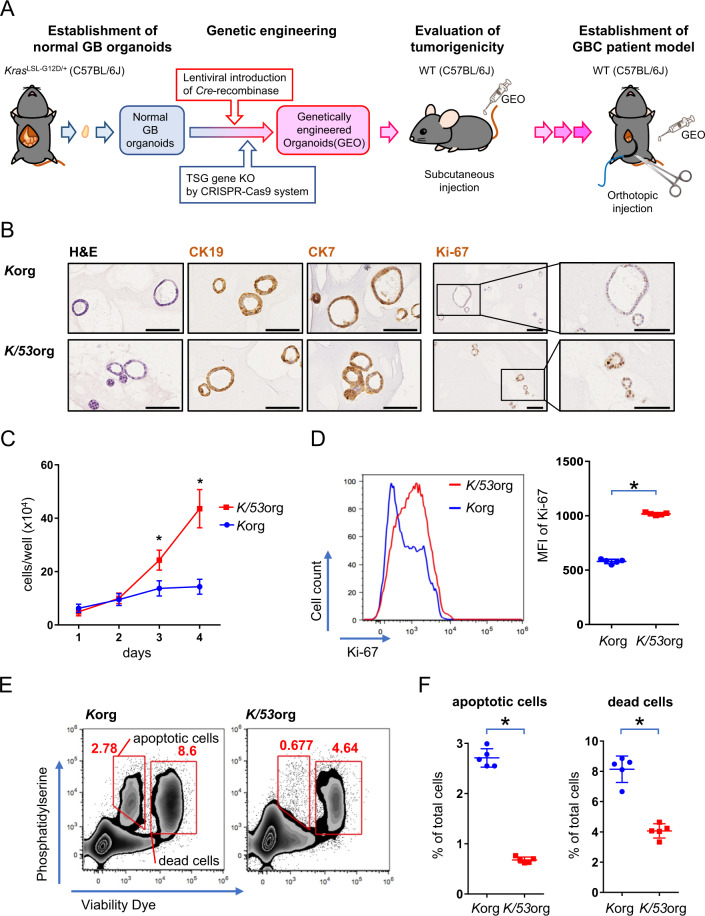
Generation of murine gall bladder (GB) organoids with Kras^G12D^ and p53 loss. **A** Schematic view of the experimental design. *TSG* tumor suppressor gene, *KO* knockout, *WT* wild type. **B** Immunohistochemical staining of organoids. *K*org, organoids expressing *Kras*^*G12D*^; *K/53*org, *K*org with *Trp53* knockout. Scale bar, 100 μm. **C** The proliferation rate in organoid culture. Mean cell count of six wells ± SD is shown. *, significant (*p* < 0.05). **D**–**F** Flow cytometry analysis of *K/53*org and *K*org at day 4 in **C**. **D** Ki-67 expression profiles. Left panel displays a representative histogram. Right panel displays a summary of the results from replicates (*n* = 5). *MFI* median fluorescent intensity. **E** Density plots of cell viability analysis. Representative images are shown. Among the phosphatidylserine^high^ population, viability dye^low^ fraction and viability dye^high^ fraction were interpreted as apoptotic and dead cells, respectively. *, significant (*p* < 0.05). **F** Summary of the results from replicates (*n* = 5) in **E**. *, significant (*p* < 0.05).

To induce GB tumors from normal cells under a thoroughly immunoproficient condition, we generated GB organoids with *Kras* activation and p53 loss (Fig. 1A). Specifically, GB organoids from *Kras*^*LSL-G12D/+*^ mice of the C57BL/6J strain were propagated in matrigel (Fig. S1A), in which *Kras*^*G12D*^ expression was induced by lentivirus-mediated introduction of *Cre* to remove the stop codon (Fig. S1B). The *Kras*^*G12D*^*-*expressing organoids (hereafter, *K*org) were transfected with a double nickase CRISPR/Cas9 vector targeting the *Trp53* gene, followed by limited dilution for single-cell cloning (Fig. S1C). We obtained a total of four clones with a microdeletion in *Trp53* that harbored an identical 10-bp frameshift deletion (Fig. S1D), confirming accurate gene editing by the double nickase. The loss of p53 protein by immunoblotting (Fig. S1E) confirmed the ablation of *Trp53* in genome-edited *K*org (hereafter, *K/53*org). Histological analysis demonstrated that both *K*org and *K/53*org comprised monolayer cells expressing the epithelial cell marker CK19 and a GB cell marker CK7; however, more Ki-67-positive cells were detected in *K/53*org, indicating higher proliferation potential (Fig. 1B). To verify the functional inactivation of p53, we selected *K/53*org clone #1 among the four clones for comparison with the bulk population of *K*org. As predicted, *K/53*org clone #1 had a significantly higher Ki-67 labeling index (Fig. 1C), proliferated more rapidly (Fig. 1D), and had fewer dead and apoptotic cells (Fig. 1E, F). Similar results were obtained for clone #2 (Fig. S1F, G), confirming the reproducibility of the results. We then cryopreserved the *K/53*org clone #1 for further analyses.

### Subcutaneous tumor development from gene-edited *K*org under intact immune system

**Fig. 2:**
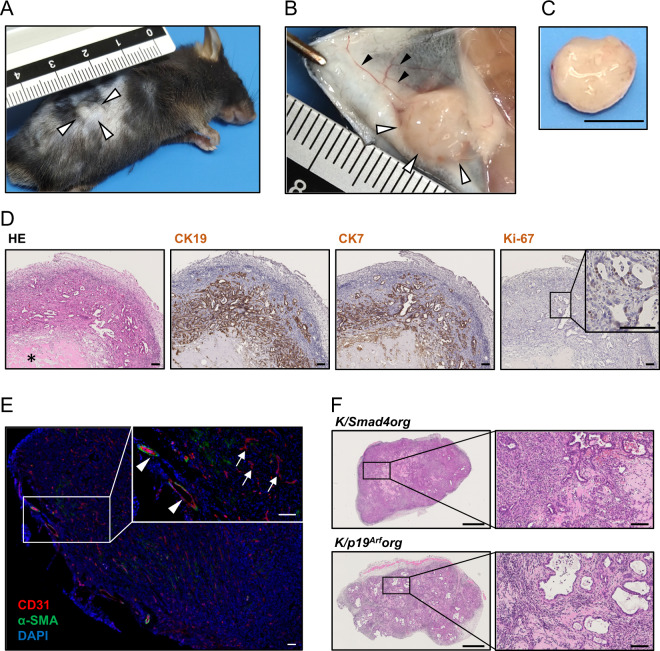
Subcutaneous tumor development from gall bladder (GB) organoids in syngeneic mice. **A** Macroscopic view of a subcutaneous tumor in a WT C57BL/6 J mouse. A representative image of a subcutaneous tumor (open arrowhead) derived from injected *K/53*org is shown. **B** Neo-angiogenesis in the tumor. Induction of surrounding blood vessels (closed arrowhead) into the tumor (open arrowhead) is shown. **C** An isolated subcutaneous tumor. Scale bar, 5 mm. **D** Histological findings and immunohistochemical staining of *K/53*org subcutaneous tumor. Note that hyalinization of the stroma (asterisk) is indicative of central necrosis. Scale bar, 100 μm. **E** Immunofluorescence staining of tumor vessels. Left, vascular endothelial cells (red, CD31) and pericytes (green, alpha smooth muscle actin [α-SMA]) are stained. The nucleus (blue) is visualized by DAPI. Scale bar, 500 μm. Right, vessels outside the tumor covered by α-SMA-positive cells (arrowhead) and uncovered vessels in the tumor (arrow). Scale bar, 100 μm. **F** Histological findings of subcutaneous tumors. K/Smad4org, Korg with Smad4 knockout. K/p19^Arf^org, Korg with p19^Arf^ knockout. Scale bar, 500 μm (low magnification), 100 μm (high magnification).

We previously documented the potent tumorigenicity of GB organoids with *Kras*^*G12D*^ expression and p53 loss, but not with *Kras*^*G12D*^ expression alone, in the subcutis of nude mice^6^. To evaluate tumorigenicity under an intact immune system, we injected *K*org and *K/53*org into the subcutaneous tissue of syngeneic WT mice. *K/53*org, but not *K*org, developed palpable tumors within several weeks in all 10 mice tested (Fig. 2A). The tumors were accompanied by prominent neo-angiogenesis (Fig. 2B), were solid in nature with a white color (Fig. 2C), and were histologically diagnosed as tubular adenocarcinoma with central necrosis (Fig. 2D) as frequently observed tumors with rapid growth. Consistent with GB cancer, the cells exhibited the expression of CK19 and CK7 and a higher Ki-67 positive rate than that of surrounding normal cells (Fig. 2D). Immunofluorescence staining revealed that the majority of the tumor vessels marked by CD31 were not covered with pericytes marked by alpha smooth muscle actin (Fig. 2E), as is commonly observed in newly formed intratumoral vasculature^15^. These results suggest that *K/53*org might be highly tumorigenic, over-riding the possible anti-tumor immune response of the syngeneic host. To ensure the applicability of this approach to p53 loss-independent carcinogenesis modeling, we re-analyzed the published data^16^ and identified 13 commonly mutated genes in GB cancer with intact *TP53* (Table S1). We selected *p19*^*Arf*^, a gene transcribed from the *Cdkn2a* locus, and *Smad4* for CRISPR/Cas9-based knockout in *K*org because a subset of human GB cancer harbors *KRAS* mutations together with *SMAD4* mutations or homozygous *CDKN2A* deletion (Table S1). We obtained four and five independent clones with identical deletions in *Smad4* (Fig. S2A) and *p19*^*Arf*^ (Fig. S2B), respectively. These clones had lost the expression of the encoding proteins (Fig. S2C, D). Upon inoculation in WT syngeneic mice, both *K/Smad4*org and *K/p19*^*Arf*^org developed subcutaneous tumors in all five cases tested for each organoid (Fig. 2F). Both gene-edited *K*org (Fig. S2E) and derived tumors (Fig. S2F) retained low levels of p53 protein expression. As these findings would exclude the possibility of p53 deletion, silencing, or accumulation by mutations, it is probable that *K/Smad4*org and *K/p19*^*Arf*^org developed GB tumors even with an active p53 pathway.

We also investigated whether other oncogenes could drive GB carcinogenesis. We selected *Pik3ca*^*H1047R*^, a commonly mutated oncogene in GB cancer (Table S1), and examined its tumorigenicity when combined with p53 loss. After verifying recombination in GB organoids from *Rosa26*-*Pik3ca*^*H1047R*^; *Trp53*^*flox/flox*^ mice (Fig. S3A), we confirmed the resultant AKT activation and p53 loss (Fig. S3B). Organoids (Fig. S3C) subcutaneously inoculated into nude mice only developed cysts in two cases tested (Fig. S3D), one of which displayed only a single tiny solid lesion that developed after longer monitoring (Fig. S3E), and the other displayed only tall epithelia with no atypia (Fig. S3F). Based on these findings, we declined further investigation on the oncogenic effects of *Pik3ca*^*H1047R*^ in GB.

### Development of an orthotopic GB cancer model by a two-step implantation approach

Next, we aimed to develop orthotopic tumor models by intra-GB injection of *K/53*org. In the abdominal cavity, GB was attached to the connective tissue (Fig. S4A). We injected *K/53*org resuspended in matrigel into the GB lumen of syngeneic mice (Fig. S4B). Despite many attempts, the matrigel did not solidify in GB after injection, allowing organoids to readily and freely flow out to the lower bile duct. To avoid leakage, we ligated the GB neck with a thread prior to injection (Fig. S4C), which retained the organoids inside the GB. However, all mice eventually died from stenosis-induced lethal cholecystitis. After all these efforts, we did not proceed with the development of an orthotopic GB cancer model using a single-step implantation approach.

**Fig. 3:**
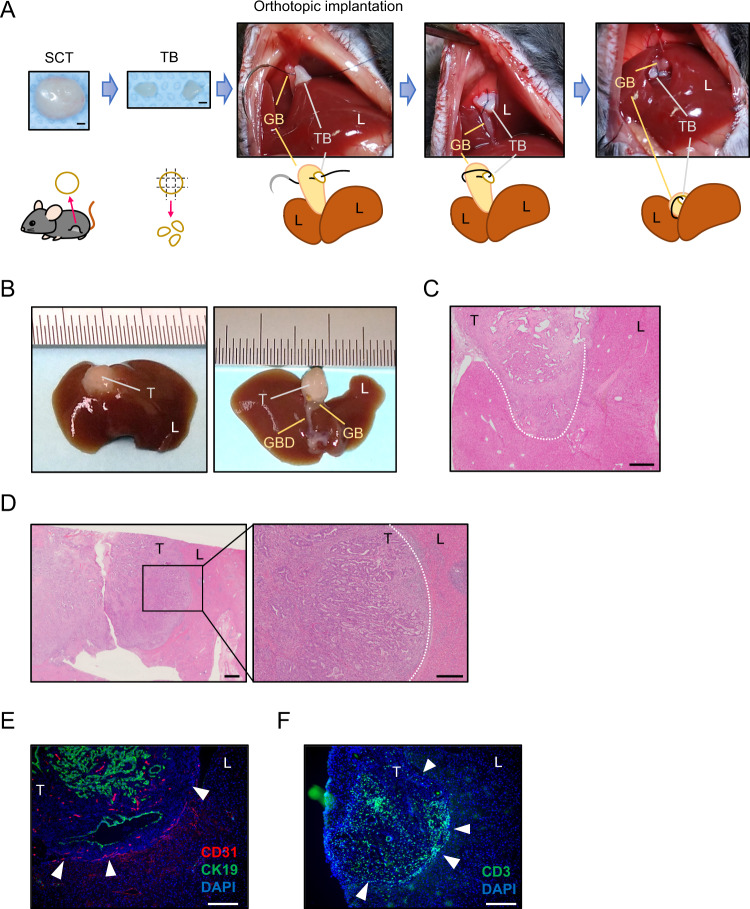
Development of an orthotopic and syngeneic gall bladder (GB) cancer model. **A** A typical workflow of the orthotopic GB cancer model. A subcutaneous tumor (SCT) was cut into 3-mm fragments (tumor bud: TB) and sutured directly to the outer surface of the GB of a C57BL/6 J mouse. Scale bar, 1 mm. L indicates the liver. **B** The macroscopic findings of an orthotopic GB cancer. Left panel, replacement of the GB by the tumor (T). Right panel, a reversed view of the GB tumor, and the common bile duct (CBD). **C** Hematoxylin and eosin (H&E) staining of the tumor. Direct invasion of cancer cells into the liver tissue (white dotted line). Scale bar, 200 μm. **D** H&E staining example of a human GB cancer is shown. The borderline of the tumor and liver is indicated by a white dotted line. Scale bar, 2 mm (left), 500 μm (right). **E** Immunofluorescence staining of the tumor. Vascular endothelial cells (red, CD31) and epithelial cells (green, CK19) are stained. Note that angiogenesis is observed in the liver tissue adjacent to the tumor surface (arrowheads). Scale bar, 200 μm. **F** Immunofluorescence staining of T cells. Note that the T cells (green, CD3) are infiltrating the tumor boundary (arrowheads) in contact with the liver. Scale bar, 200 μm.

While developing an organoid-based orthotopic model for pancreatic cancer in syngeneic mice, we experienced failures with a single-step implantation approach. This issue was overcome by the initial generation of subcutaneous tumors in nude mice, followed by orthotopic implantation of tumor fragments into syngeneic mice^7^. Thus, we adopted a two-step implantation approach. We first generated *K/53*org-derived subcutaneous tumors in syngeneic mice. The tumors were subsequently minced into 2-mm fragments, designated as the “tumor bud (TB)” (Fig. 3A). A TB was then inserted into the GB with the tip cut down followed by fine suturing (Fig. S4D). Although the TB remained inside the GB without inducing cholecystitis, tumors did not develop, presumably owing to the digestion of the TB by bile (Fig. S4E). To avoid direct exposure to bile, we fixed the TB outside the GB by suturing (Fig. 3A). This method, which we designated as the “implantation of organoid-derived TB (IoTB),” proved effective and allowed the transplanted TBs to grow into solid tumors (Fig. 3B). The induced GB tumors were diagnosed as moderately differentiated adenocarcinoma with liver invasion (Fig. 3C). Consistent with GB cancer, the tumors were CK19- and CK7-positive (Fig. S4F) and had a higher Ki-67-positive rate than that of normal GB cells (Fig. S4G). Notably, the tumors histologically resembled human GB cancer (Fig. 3D). In the liver tissue adjacent to the tumors, prominent neo-angiogenesis (Fig. 3E) and enrichment of tumor-infiltrating T cells (Fig. 3F) were observed at the tumor boundary, as commonly observed in human GB cancer.

### Positive correlation of orthotopic tumors with human cancer in the transcriptome

**Fig. 4:**
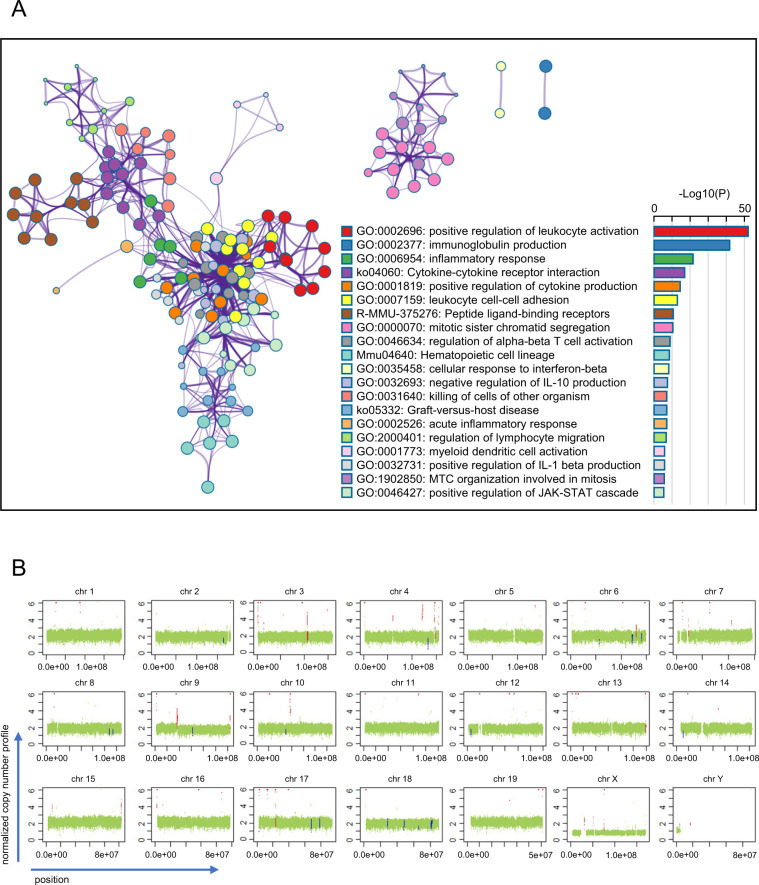
Gene expression profiles and genetic instability of induced gall bladder (GB) cancers. **A** RNA-seq analysis using the orthotopic GB cancer tissues, GBCa-#1, #2, and #3, and normal GB tissues, GB-#1, #2, #3. The network layout of pathway enrichment analysis is shown. The size of each circle node is proportional to the number of input genes that fall under that term. Terms with a similarity score >0.3 are linked by an edge. Top 20 statistically enriched terms are shown with their *p* value. Log10(P), the *p* value in log base 10. *MTC* microtubule cytoskeleton. *IL* interleukin. **B** Summary of genome instability. Gene copy number alteration was calculated based on whole-genome analysis of GBCa-#1 is shown. Green, red and blue dots indicate base line, gain, and loss, respectively. *Chr* chromosome.

To better characterize the orthotopic tumors, RNA-sequencing (RNA-seq) analysis of GB tumors and normal GBs was performed. To maximize the transcriptional signature, the three largest tumors (GBCa-#1, -#2, and -#3) among 10 tumors derived from simultaneously inoculated TBs were selected. In total, 5838 genes were differentially expressed by more than twofold in tumor tissues than that in normal GBs (Fig. S5A, Table S2), thus displaying high similarity between the tumor and normal groups (Fig. S5B). Pathway enrichment analysis based on genes upregulated by more than tenfold in tumor tissues identified multiple pathways related to immunoregulation and mitosis as clusters (Fig. 4A), as is generally observed in cancer. We performed the same analysis using previously reported RNA-seq data from human GB cancer and normal tissues^17^. Based on the size and number of nodes, human GB cancer tended to exhibit higher diversity among tumors (Fig. S6A). Comparison of GB cancer between humans and the murine orthotopic model using 532 differentially expressed genes (DEGs) in both data sets showed a moderate positive correlation (*r* = 0.33, *p* < 0.0001; Fig. S6B), confirming their similarity at the transcriptome level.

Chromosomal instability is a hallmark of p53-inactivated cancer at an advanced stage. As GBCa-#1 was the largest and p53-null tumor, we evaluated its genome-wide copy number variation based on the assumption that it might harbor such prominent alterations. However, the chromosome number was stable. Nonetheless, focal copy number alterations were detected at multiple loci throughout the genome, indicative of genetic instability (Fig. 4B). These amplicons and deletions did not contain major known cancer-related genes (Table S3).

### Similar kinetics of the immune cell profile in orthotopic and subcutaneous tumors

**Fig. 5:**
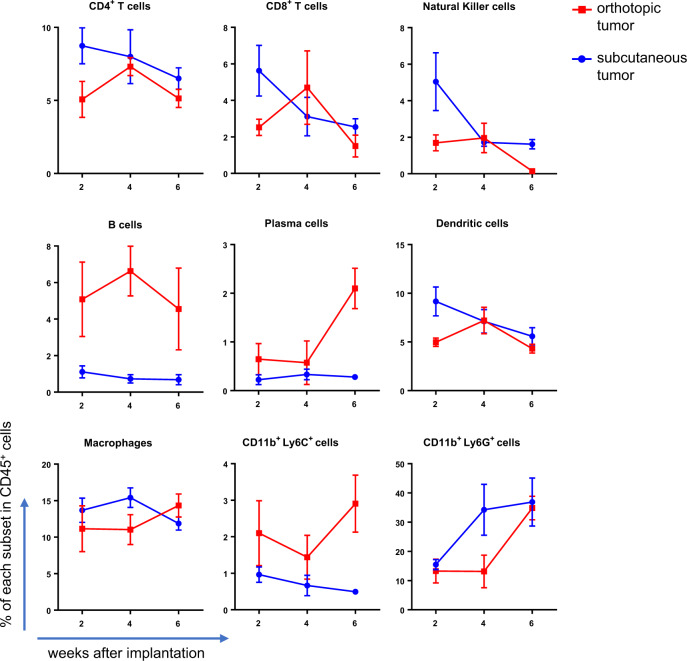
Time-course profiling of TICs. Flow cytometry analysis of TICs. The proportion of each subset of immune cells to CD45-positive cells is shown. Data were obtained at 2, 4, and 6 weeks after implantation (*n* = 5).

To investigate how the tumor microenvironment could vary by location and change over time, flow cytometry-based time-course profiling of tumor-infiltrating immune cells (TICs) was performed after subcutaneous inoculation of organoids and IoTB. Representative subsets of TICs were successfully detected (Fig. S7), which revealed that the proportion of CD4^+^ T cells, CD8^+^ T cells, natural killer cells, and dendritic cells gradually decreased in both models (Fig. 5). Although the proportion of B cells did not change in either model, the proportion of plasma cells increased at 6 weeks after implantation in the orthotopic model. The proportion of macrophages and CD11b^+^ Ly6C^+^ cells remained relatively constant, whereas that of CD11b^+^ Ly6G^+^ cells increased by threefold at 6 weeks after implantation in both models, although the increase was already evident for subcutaneous tumors at an earlier time point (Fig. 5). Collectively, the TIC profile in each condition appeared similar, and its partial change in response to local implantation of cancer cells might also be similar.

### Prospective evaluation of drug sensitivity using the orthotopic GB cancer patient model

**Fig. 6:**
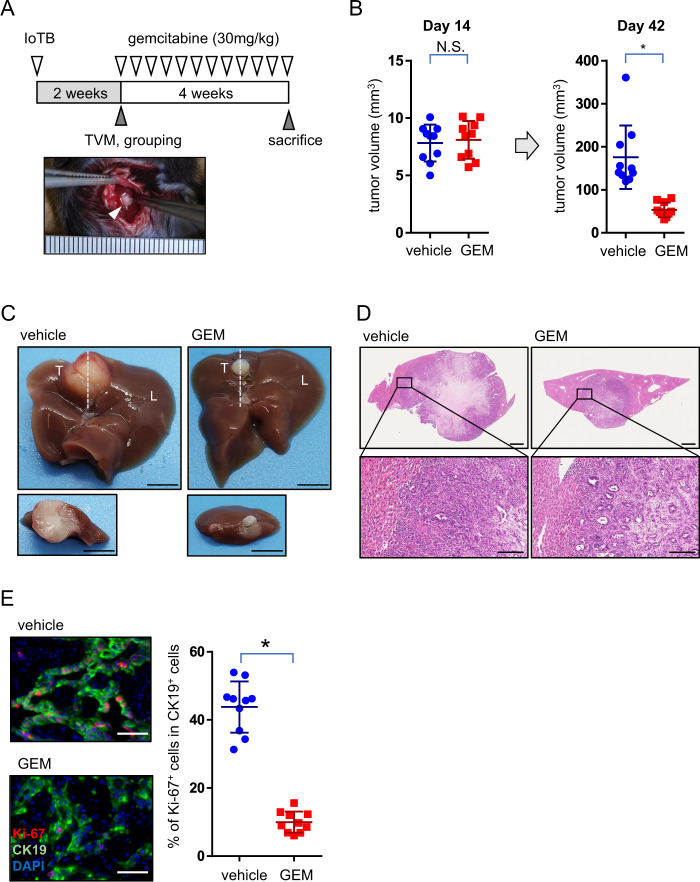
Chemotherapeutic treatment in orthotopic and syngeneic gall bladder (GB) cancer model. **A** A scheme of chemotherapeutic treatment. *TVM* tumor volume measurement. An inspected tumor is shown (open arrowhead). **B** The tumor volumes in vehicle- (*n* = 10) or gemcitabine-treated groups (*n* = 10) on day 14 and 42 after IoTB. N.S., not significant. *p < 0.0001. **c** Macroscopic findings of the treated tumors. Representative images are shown. *T* tumor, *L* liver. Dashed lines (upper panel) indicate the cut surface of the tumors (lower panel). Scale bar, 5 mm. **D** Microscopic features of the tumors. Representative images of hematoxylin and eosin (H&E) staining are shown. Upper panel, whole images of the tumors. Scale bar, 1 mm. Lower panel, invasion front adjacent to the liver. Scale bar, 0.1 mm. **E** The proportion of Ki-67^+^ proliferating cells among CK19^+^ epithelial cells in each group. Left, representative images of immunostaining. Cell proliferation (red, Ki-67), epithelial cells (green, CK19), and the nucleus (blue, DAPI) are visible. Scale bar, 50 μm. Right, summary of the results. *p = 0.036.

To verify the preclinical relevance of the orthotopic GB cancer patient model, we administered gemcitabine (Gem), a standard chemotherapeutic agent for human GB cancer. *K/53*org clone #1 was subcutaneously inoculated into syngeneic mice. Tumors developed in all 10 mice tested. The tumors were harvested within 4 weeks after inoculation when the tumor reached 1-cm diameter. Each tumor was minced into four pieces. A total of 40 TBs were shuffled and separately cryopreserved. To prepare a cohort of 20 mice, we randomly thawed 20 TBs and performed IoTB on day 0. For accurate prospective evaluation of therapeutic efficacy, the tumor volume was measured by open inspection of the abdomen on day 14, immediately before initiation of the Gem treatment. The cohort was divided into two groups with equal tumor burden (Fig. 6A). Intraperitoneal Gem treatment at a dose of 30 mg/kg three times a week for 4 weeks significantly inhibited tumor growth (Fig. 6B). Although complete tumor clearance was not achieved, tumor growth was significantly inhibited in the Gem-treated group (Fig. 6C), in contrast to tumors of the vehicle group accompanied by significant central necrosis (Fig. 6D). Consistent with this observation, the Ki-67 labeling index of the epithelial cells was significantly lower in the Gem-treated group than in the vehicle group (Fig. 6E). These observations suggest that this model might be suitable for preclinical studies in syngeneic mice.

## Discussion

As patient-derived cancer models, xenografts in immunodeficient mice and organoids in culture have recently drawn attention as promising modalities for preclinical studies owing to their advantages over cancer cell lines that may have undergone extensive alterations from original tumors^18^. In particular, retention of gene mutations, tumor heterogeneity, and cellular differentiation in these two models, along with the presence of stromal cells in xenografts or the 3D structure of organoids, may allow a more accurate prediction of the therapeutic response in patients^19,20^. However, they still lack an intact immune system, which could potentially modify tumorigenesis^21^ and therapeutic responses to cytotoxic agents and immune checkpoint inhibitors^16^, providing an opportunity for the development of an ideal model. In this study, we prioritized the integration of the inherent immune system to a novel model, thereby excluding patient-derived xenografts (PDX) or organoids. As in vivo models, two GEMs for GB cancer have been previously documented, although they lacked sufficient tissue specificity for gene recombination. One is a transgenic mouse for rat *ErbB2* under the control of the bovine keratin 5 promoter, which coincidentally developed GB cancer^9^, in addition to skin tumors, as initially intended^22^. Although the effect of chemotherapy on GB cancer was later evaluated using the same model^23^, the interpretation of the results required careful consideration because the therapeutic impact could be modified by ectopic tumors and associated immune responses. The other is a mouse carrying *Ah-Cre; Kras*^*LSL-G12D/+*^*; Pten*^*flox/flox*^, in which compound mutations were locally induced by the intraperitoneal injection of a chemical, leading to tumor development in the whole biliary system^10^. Collectively, GB-specific gene recombination is yet to be established unless novel *Cre* transgenic mice are developed.

Instead, we achieved tissue specificity by physically isolating GB from adult mice, followed by organoid culture and lentiviral gene transfer. Using the IoTB approach, orthotopic tumor development was achieved under physiological immune system and in a situation where surrounded by genetically normal cells. Thus, our model would mostly satisfy the aforementioned criteria set for an ideal cancer model. Moreover, it has additional technical advantages. First, genetic aberrations were serially introduced into the organoids. This procedure enabled the direct comparison of the tumorigenic impact of each gene modification in identical organoids with the same genetic background. Second, the two-step tumor development facilitated the rapid development of orthotopic tumors in a highly scalable manner. The IoTB procedure is simple and can be completed in ~5 min per mouse, allowing the preparation of 20–40 mice in one session without intercrossing multiple GEMs. Considering the high relevance of large cohorts in preclinical studies and immunological analyses, this scalability may be the most important aspect of these types of analyses. Third, the local anti-tumor immune response could be recapitulated by sporadic tumor development in adult immunoproficient mice. Some immune cells, such as tissue-specific macrophages, emerge during the fetal period and remain in local tissues throughout life^24,25^. Hence, even genetic engineering specific to tissue epithelial cells may indirectly affect the local immune response to tumors, as long as it is conducted in the embryonic stages. We demonstrated that the profiles and kinetics of TICs in both subcutaneous and orthotopic tumors were mostly similar, justifying the temporal use of subcutaneous tissue as a surrogate for GB before IoTB. We also demonstrated that CD11b^+^ Ly6G^+^ cells, which could contain both neutrophils and polymorphonuclear myeloid-derived suppressor cells^26^, significantly increased over time in both tumors. Considering each fraction may have distinct functions in normal tissues and neoplastic tissues^27^, our models might help dissect anti-tumor immune responses and elucidate the roles of these TICs in tumors.

We provided histological and gene expression profile data concerning the similarity between human GB cancer and orthotopic mice models. Specifically, DEGs in both human and mouse data sets showed a moderate positive correlation, although gene symbols and the functions of each gene were not completely identical among different species. In addition, human GB cancer data did not contain any information on the mutation status in each tumor^17^, whereas orthotopic tumors stereotypically harbored compound mutations in *Kras* and *Trp53*. These results could justify the relevance of this method in establishing GB cancer patient-derived models and suggest that additional data on human GB cancer samples could potentially improve the correlation between human and mouse tumors. The questions of when and how these amplifications were acquired and selected remain. Given the highest proliferation rate of *K/53*org in serum-free optimized culture conditions, acquisition of genetic alterations would not possibly lead to the overwhelming of the whole population. We speculate that such genetic alterations were established during tumor development after implantation owing to a short supply of growth factors, as we previously observed in an organoid-based pancreatic carcinogenesis model^7^. It would be interesting to investigate whether serial transplantations could lead to chromosomal instability.

We termed the approach used herein as IoTB based on conceptual similarity to the reconstitution of liver tissues by inoculation of the liver bud, an aggregate of hepatocytes induced from induced pluripotent stem cells, endothelial cells, and mesenchymal stem cells^28,29^. Although we repeatedly confirmed that the two-step process was necessary to establish a syngeneic orthotopic GB cancer model, a previous study documented an orthotopic xenograft model of human GB cancer cells by a single-step approach^30^. Contrary to our observation, injection of Mz-ChA-1 cells mixed with matrigel into the GB lumen reportedly resulted in the rapid solidification of matrigel and achieved a 100% tumor take rate without any interference by bile. The reason for the irreproducibility in our study remains unclear. However, based on the figures presented in their study, we suspect that cells might have been injected into the liver capsule. Alternatively, cross-species differences in the immune response could affect the tumor take rate. Further studies are required to address this issue.

Unlike major cancers, clinical trials targeting biliary tract cancer have been conducted for multiple sites altogether, including the intrahepatic or extrahepatic bile duct, GB, and ampulla^31^. However, the first clinical trial with only GB cancer patients was reported in 2010^32^. These situations highlighted the need for a modality to assess the effect of chemotherapy in a GB cancer-specific manner; this was performed using Gem in this study and may be theoretically applicable to immune checkpoint inhibitors. The next challenge will be the development of conceptually similar models with patient-derived GB cancer organoids or genetically engineered human GB organoids. However, we did not extend the scope of this study in this direction for several reasons. First, a high take rate would not be expected for patient-derived tumor organoids, considering the low take rate of PDX for human biliary cancer^33^. Second, human organoids might be refractory to transformation by modification in 2–3 genes based on the low take rate exhibited in similar studies in other organs^3^. Third, an intact immune system is absent as long as immunodeficient mice are used. Future development of a co-culture system of organoids and immune systems or tumor development in humanized mice will be necessary to address this issue.

In conclusion, we developed a novel organoid- and allograft-based syngeneic GB cancer model that could provide an ideal platform for preclinical studies. The generality of our approach in the development of a fine-tuned patient model will accelerate drug discovery and the implementation of precision medicine in many types of cancer.

## Materials and methods

Detailed information is provided in the Supplementary Materials and Methods.

### Mice

The *Kras*^*LSL-G12D/+*^ mice, *Trp53*^*flox/flox*^, and *R26-Pik3ca*^*H1047R*^ mice were imported from The Jackson Laboratory (Bar Harbor, ME) and were maintained in a C57BL/6J background. These mice were intercrossed to generate compound mutant mice.

### Organoid culture and lentiviral infection

Primary GB cells were obtained as previously described^6^. Organoid culture and lentiviral infection were performed as previously described^4^. LV-*Cre* pLKO.1 (Addgene plasmid 25997) was used for lentiviral infection. Genotyping and confirmation of *Cre-*mediated recombination were performed as previously described^6,34^.

### Gene editing by CRISPR-Cas9

For CRISPR-Cas9 vectors, pre-designed double nickase plasmids for *Trp53*, *Smad4*, and *p19*^*Arf*^ (Santa Cruz Biotechnology, Dallas, TX) were used.

### Western blotting

GB organoids were harvested after lysis of matrigel with Cell Recover Solution (BD Biosciences, Franklin Lakes, NJ) for 1 h on ice. Western blotting was performed according to the standard protocol.

### Cell proliferation assay

GB organoids were seeded at a concentration of 1 × 10^4^ cells per well in a 24-well plate and cultured using the same procedures for a subculture (day 0). From day 1 to day 4, organoids in each well were harvested and dissociated with Accumax (Innovative Cell Technologies Inc., San Diego, CA) for 20 min at 37 °C.

### Flow cytometry

Cells were stained with fluorescein-labeled monoclonal antibodies at 4 °C in fluorescence-activated cell sorting staining buffer (Hanks' Balanced Salt Solution with 0.5% bovine serum albumin (BSA) and 0.01% sodium azide) after blocking CD16/CD32 (clone 93; BioLegend, San Diego, CA). To detect apoptotic and dead cells, anti-phosphatidylserine antibody (clone 1H6, Merck Millipore, Burlington, MA) and Zombie NIR™ Fixable Viability Dye (BioLegend) were used according to the manufacturer’s protocol.

### Subcutaneous and orthotopic tumor development

Approximately 5 × 10^5^ cells were resuspended in 140 μL of matrigel and subcutaneously injected into WT C57B/L6 mice. For orthotopic implantation, TBs were thawed on the day of transplantation and sewed on the outer surface of the GB using a 6–0 nylon suture.

### Histological analysis

For immunofluorescence staining, tissues were fixed and embedded in low-melting-point paraffin using a HOPE^®^ Kit (Polysciences, Warrington, PA), following the manufacturer’s instructions. A board-certified pathologist (M.E.) reviewed the histological features of the tumors. As a reference, anonymous hematoxylin and eosin-stained slides of human GB cancer were provided by the Department of Pathology, Yokohama City University, Yokohama City, Japan.

### RNA-seq

RNA-seq was performed at TaKaRa Bio. Inc. (Shiga, Japan). Read mapping on a genomic sequence was performed with DRAGEN Bio-IT software ver3.6.3 (Illumina, San Diego, CA). The count data were analyzed using the tag count comparison (TCC) R package^35^ through TCC-Graphical User Interface^36^. The basic algorithm of TCC was previously described^37^.

### Data analysis of human GB cancer gene expression profile

RNA-seq data for human GB cancer and matched normal GB tissues were obtained from a previous report^17^. Using a set of genes that were upregulated by ≥10-fold in the GB cancer group than in the normal GB tissues, we performed further pathway analysis in the same manner as with the mice data.

### Whole-genome sequencing

Whole-genome sequencing was performed at TaKaRa Bio. Inc. The sequencing library was prepared using TruSeq DNA PCR-Free Library Prep Kit (Illumina) and IDT for Illumina TruSeq DNA UD Indexes (Illumina), following the manufacturer’s instructions.

### In vivo chemotherapeutic treatment

Two weeks after IoTB, the abdomen was temporarily opened and the tumor diameter was measured. The tumor volume (*V*) was calculated according to the following formula: *V* (mm^3^) = 1/2 × width (mm) × width (mm) × length (mm). Twenty mice were divided into two groups so that the average tumor volume was approximately the same. Gem (Sigma-Aldrich, St. Louis, USA, 30 mg/kg body weight) or a vehicle (phosphate-buffered saline) was intraperitoneally administered three times a week for 4 weeks.

### Statistical analysis

The data were analyzed using the nonparametric Mann–Whitney *U* test with GraphPad Prism 6 software (version 6.0f; GraphPad, San Diego, USA). Data were considered significant at *p* < 0.05.

## Supplementary information

Supplementary Materials and Methods

Supplementary Figures and Tables

## Data Availability

Nucleotide sequence data reported are available in the DDBJ/EMBL/GenBank databases under the accession numbers DRA011540 and DRA011541.
