# Supplementary material for: Precision modeling of gall bladder cancer patients in mice based on orthotopic implantation of organoid-derived tumor buds
Source: Oncogenesis. 2021 Apr 17;10(4):33. doi: 10.1038/s41389-021-00322-1 (PMC8053198; doi:10.1038/s41389-021-00322-1)
Supplement: Supplementary file 1 — Supplementary Materials and Methods [file 41389_2021_322_MOESM1_ESM.docx]

**SUPPLEMENTARY MATERIALS AND METHODS**

**Mice**

C57BL/6J wild type (WT) mice and immune-deficient nude BALB/cAnu/nu mice were purchased from CLEA Japan Inc. (Tokyo, Japan). Animal studies were conducted according to the Guidelines for Animal Experiments provided by the Committee for Ethics in Animal Experimentation of Yokohama City University and Chiba Cancer Center, which meets the ethical standards required by law and follows the guidelines for animal experimentation in Japan.

**Organoid culture**

GB was isolated from C57BL/6J mice at 6 weeks of age. After thoroughly washing bile from the GB with cold phosphate-buffered saline (PBS), the tissues were chopped up as small as possible on ice. Tissues were digested with 2 U/mL of dispase II (Sigma-Aldrich, St. Louis, MO) and 1 mg/mL collagenase P (Sigma-Aldrich) for 30 min at 37 °C. The digested tissues were washed with cold PBS and resuspended in organoid culture media: Advanced DMEM/F12 (Thermo Fisher Scientific, Waltham, MA) supplemented with L-glutamine (Thermo Fisher Scientific), penicillin-streptomycin (Thermo Fisher Scientific), mouse epidermal growth factor (Peprotech, Cranbury, NJ), Noggin (Peprotech), Jagged-1 (AnaSpec, Fremont, CA), Y27632 (Wako, Osaka, Japan), and R-spondin1 (R&D Systems, Minneapolis, MN). A 12-well plate was coated with 60 µL of Matrigel (Corning, Corning, NY) and incubated for 30 min at 37 °C to ensure solidification. Resuspended cells were seeded on polymerized Matrigel and incubated overnight at 37 °C (day 0). To avoid the digestion of Matrigel by GB-derived proteases, 10% fetal bovine serum was added only on day 0. The next morning (day 1), the floating dead cells and media were aspirated. The viable cells attached to the Matrigel were sandwiched between 80 µL Matrigel. After the Matrigel was polymerized, 800 µL/well of organoid culture media was overlaid.

**Lentiviral infection**

Organoids were harvested along with Matrigel using a small cell scraper. After centrifugation at 500 × *g* for 5 min at 25 °C, the supernatant was aspirated. The pellets were digested in Accumax (Innovative Cell Technologies Inc., San Diego, CA) for 15 min at 37 °C. After incubation, the organoid cells were washed with Dulbecco’s PBS and resuspended in organoid medium. Dissociated cells were seeded on polymerized Matrigel in a 12-well plate and incubated for 2–4 h at 37 °C. After confirming that the viable cells were attached to the Matrigel, the floating cells and media were aspirated. The cells were sandwiched between 80 µL of Matrigel per well. After the Matrigel was polymerized, 800 µL/well of organoid medium was added. For lentiviral infection, 1 × 10^5^ single cells were resuspended in 500 μL of 1.5× culture medium supplemented with 3.75 μL of Transdux (System Biosciences, Palo Alto, CA) and mixed with 250 μL of 10-fold concentrated viral particles. The cells were then seeded on polymerized Matrigel in a 12-well plate and incubated overnight at 37 °C, followed by the same procedures for subculture.

**Gene editing by CRISPR-Cas9**

A total of 1 × 10^4^ singly dissociated GB cells were seeded on polymerized Matrigel in a 24-well plate. Lipofectamine^®^ 3000 (Thermo Fisher Scientific) with CRISPR-Cas9 vectors was added according to the manufacturer’s protocol. For single-cell cloning, transduced organoid cells were seeded at a very low density, and the resultant organoids grown from a single cell were collected under a microscope. After overnight incubation at 37 °C, a limited dilution was performed for single-cell cloning. Genotyping of targeted genes by CRISPR-Cas9 vectors in each clone was performed by polymerase chain reaction-based amplification of the target regions in the vector, followed by direct sequencing of the resultant amplicon. The target regions of the pre-designed double nickase plasmids for Trp53 were 5′- gaagtcacagcacatgacgg -3′ and 5′- agatggccatggcgcggaca -3′. The regions for Smad4 were 5′- aacggagacgtacagcgccc -3′ and 5′- cgcgattacttggcgggtgt -3. The regions for p19Arf were 5′- tcgtgcgatcccggagaccc -3′ and 5′- aagaaaaccctctcttggag -3′. The sequences of the primers used for genotyping after gene editing by the CRISPR-Cas9 vectors for Trp53 were 5′- gaagtcacagcacatgacgg -3′ and 5′- agatggccatggcgcggaca -3′. The primers for Smad4 were 5′- ccgtccttacccactgaagg -3′ and 5′- ccacaaaagctccaagtggc -3′. The primers for p19Arf were 5′- ggttcttggtcactgtgagg -3′ and 5′- agcttcggagggcctttc -3′.

**Western blotting**

The organoids were lysed in M-PER® buffer (Thermo Fisher Scientific). Protein concentrations were quantified using a DS-11 spectrophotometer (Denovix, Wilmington, DE). The protein solution was mixed with a triple amount of 4× sample buffer (0.25 M Tris-HCl pH 6.8, 0.02% bromophenol blue, 8% sodium dodecyl sulfate, 40% glycerol, 10% 2-mercaptoethanol) and heated at 100 °C for 5 min. Electrophoresis was performed using 10 μg of the protein. The proteins were electrically transferred to a polyvinylidene difluoride (PVDF) membrane in a wet tank. After blocking with PVDF blocking reagent for Can Get Signal® (Toyobo, Tokyo, Japan), the membranes were incubated overnight at 4 °C with primary antibodies. Primary antibodies against p53 (#32532, Cell Signaling Technology, Danvers, MA), p19Arf (5-C3-1, Santa Cruz Biotechnology, Dallas, TX), Smad4 (EP618Y, Abcam, Cambridge, UK), p-AktS473 (#4060, Cell Signaling Technology), β-actin (#4970, Cell Signaling Technology), and glyceraldehyde-3-phosphate dehydrogenase (#5174; Cell Signaling Technology) were used. The membranes were further incubated with horseradish peroxidase (HRP) conjugated anti-rabbit secondary antibody for 1 h at 25 °C and visualized using Immobilon Forte Western HRP substrate (Merck Millipore, Burlington, MA).

**Cell proliferation assay**

Cells were stained with 0.4% trypan blue solution (Thermo Fisher Scientific), and viable cells were counted based on the exclusion of the dye. Cell proliferation was compared between GB-organoid cells with KrasG12D and *Kras^G12D^; Trp53^-/-^*. For each group, six wells were counted each day. The experiments were performed in duplicate.

**Flow cytometry**

The antibodies used for staining were specific for mouse CD45 (clone 104), CD3ε (clone 145-2C11), CD4 (clone GK1.5), CD8α (clone 53-6.7), NK1.1 (clone PK136), CD45R/B220 (clone RA3-6B2), CD138 (clone 281–2), CD19 (clone 1D3), CD11c (clone N418), CD11b (clone M1/70), F4/80 (clone BM8), MHC-II (clone AF6-120.1), Ly6C (clone HK1.4), and Ly6G (clone 1A8) (all from BioLegend, San Diego, CA). After staining and washing, cell fluorescence was measured on an LSR II flow cytometer (BD Biosciences, Franklin Lakes, NJ) and analyzed using FlowJo software (BD Biosciences). The surface markers of each immune cell subset were as follows: CD4^+^ T cells: CD45^+^ CD3ε^+^ CD4^+^; CD8^+^ T cells: CD45^+^ CD3ε^+^ CD8^+^; NK cells: CD45^+^ CD3ε^-^ NK1.1^+^; B cells: CD45^+^ CD3ε^-^ B220^+^; plasma cells: CD45^+^ B220^-^ CD138^+^; dendritic cells: CD45^+^ CD11b^-^ CD11c^+^ MHC-II^+^; macrophages: CD45^+^ CD11b^+^ F4/80^+^ MHC-II^+^; CD11b^+^ Ly6C^+^ cells, CD45^+^ CD3ε^-^ CD19^-^ NK1.1^-^ MHC-II^-^ CD11b^+^ Ly6C^+^; and CD11b^+^ Ly6G^+^ cells, CD45^+^ CD3ε^-^ CD19^-^ NK1.1^-^ MHC-II^-^ CD11b^+^ Ly6G^+^.

**Subcutaneous and orthotopic tumor development**

Before injection, the viability of organoid cells was confirmed to be higher than 90% using trypan blue solution (Wako). Four weeks after subcutaneous inoculation of GB organoids, the developed subcutaneous tumor was harvested and cut into 2-mm pieces, which were temporarily cryopreserved at -80 °C in BAMBANKER® (GC Lymphotec Inc., Tokyo, Japan). For orthotopic implantation, a 6-week-old mouse was anesthetized by inhalation, and the abdomen was opened. The peritoneum was sutured with a 6–0 nylon suture, and the skin was closed with surgical clips.

**Histological analysis**

The organoids were embedded in iPGel (GenoStaff, Tokyo, Japan) according to the manufacturer’s instructions. Both organoids and tumor tissues were fixed in 10% formaldehyde neutral buffer solution (Nacalai Tesque, Inc., Kyoto, Japan) and embedded in paraffin. For HOPE®-fixed tissues, the paraffin blocks were sliced to a thickness of 3 μm, and the sections were stretched in a water bath (35 °C). The sections were then lifted out with a coated slide and dried in an incubator at 50 °C for 30 min. Dried sections were stored at 4 °C. Dewaxing, rehydration, and hematoxylin and eosin (H&E) staining of the HOPE-fixed tissue sections were performed according to the manufacturer’s instructions. Briefly, slides were placed in isopropanol (60 °C) for 10 min for deparaffinization and were incubated twice for 10 min in 70% cold acetone on ice for rehydration. For H&E staining, the rehydrated sections were incubated with hematoxylin (Mayers Hematoxylin, Wako) for 2–4 min. After washing with deionized water, the stained sections were incubated in eosin alcohol solution (Wako) for 2–4 min. Sliced sections (3 μm) were deparaffinized and rehydrated using xylene and ethanol. Antigen retrieval was performed by incubating the slides in HistVT One solution (Nacalai Tesque) for 20 min at 90 °C. Endogenous peroxidase activity was blocked by incubating the slides in methanol containing 0.3% hydrogen peroxide for 20 min at 25 °C. Slides were stained with primary antibodies after blocking with G-Block (GenoStaff). The antibodies used for staining were specific for mouse cytokeratin 19 (ab133496, Abcam), cytokeratin 7 (EPR17078, Abcam), Ki-67 (SolA15, Thermo Fisher Scientific), and p53 (21891-1-AP, Proteintech). After washing with PBS, HRP-conjugated secondary antibodies (Nichirei Biosciences, Tokyo, Japan) were added to the slides and incubated for 30 min at 25 °C. After washing with PBS, the binding of the primary antibodies was visualized using 3,3' diaminobenzidine as the substrate. Nuclei were stained with hematoxylin as a counterstain. The antibodies used for staining were specific for mouse CD31 (clone MEC 13.3, BioLegend), CD3 (ab5690, Abcam), α-smooth muscle actin (clone 1A4, Thermo Fisher Scientific), Ki-67 (SolA15, Thermo Fisher Scientific), and CK19 (ab133496, Abcam). For anti-CD3 and CK19 antibodies, anti-rabbit secondary antibodies were used for detection. Fluorescence images were analyzed using a BZ-9000 fluorescence microscope (Keyence, Osaka, Japan). For the analysis of Ki-67+ cells, the number of Ki-67+ and Ki-67- cells in CK19+ cells was manually counted in five microscopy fields. The average value of the counts of the five fields was taken as the value of the sample. Immunohistochemistry was performed according to the standard protocol.

**RNA-sequencing**

The tumor tissue was harvested 4 weeks after orthotopic implantation. As a control, untreated GB tissue was harvested from 10-week-old WT mice. Total RNA was purified from harvested tissues using NucleoSpin® RNA (TaKaRa Bio Inc., Shiga, Japan), according to the manufacturer’s instructions. The quality of the RNA samples was verified using an Agilent 2100 Bioanalyzer (Agilent, Santa Clara, CA). cDNA from total RNA and the sequencing library from the cDNA were prepared using SMART-Seq® v4 Ultra Low Input RNA Kit for Sequencing (Clontech Laboratories, Inc., Mountain View, CA), Nextera XT DNA Library Preparation Kit (Illumina, San Diego, CA), and Nextera XT Index Kit v2 Set A/B/C/D (Illumina) following the manufacturer’s instructions. Sequencing analysis was performed using NovaSeq 6000, NovaSeq 6000 S4 reagent kit, and NovaSeq Xp 4-Lane Kit (Illumina) following the manufacturer’s instructions.

**Data analysis of GB cancer transcriptome**

Tag count comparison computation was performed with the following parameters: normalization method, TMM; differentially expressed gene (DEG); identification method, edge R; number of iterations, 3; false discovery rate (FDR) cut-off, 0.1; and elimination of potential DEGs, 0.05. To visualize the results, a Volcano plot was generated with the following parameters: log2 (fold change) cut-off: -1 and 1 and *p*-value cut-off: 0.05. Additionally, a heat map was generated with the following parameters: selection of genes by FDR cut-off: 0.01; source: normalized; distance measure: Euclidean; agglomeration method: complete; scaling: by row (gene); and log (1+x) Transform: performed. Using a set of genes that were 10-fold (mouse)/2-fold (human) or more upregulated in the GB cancer group than in the normal GB group, we performed further pathway analysis with Metascape ^1^ with the following parameters: minimum overlap, 3; *p*-value cut-off, 0.01; minimum enrichment, 1.5; and included pathways, Gene Ontology biological processes, reactome gene sets, and Kyoto Encyclopedia of Genes and Genomes pathway. After identifying all statistically enriched terms, a subset of representative terms from this cluster was converted into a network layout. The network was visualized with Cytoscape (v3.1.2) with “force-directed” layout and with edges bundled for clarity. In the figure, each circle node represents each term and the size is proportional to the number of input genes that fall into that term. Terms with a similarity score > 0.3 are linked by an edge (the thickness of the edge represents the similarity score).

**Correlation analysis of DEGs in human and mouse GB cancer tissues**

The fold-change values of 532 genes that commonly vary between human and mouse RNA sequence datasets were obtained. Pearson’s correlation analysis was performed to analyze the correlation between them.

**Whole-genome sequencing**

The tumor tissue was harvested 4 weeks after orthotopic implantation. Genomic DNA was purified from the tissue using NucleoSpin® Tissue (TaKaRa Bio. Inc.) according to the manufacturer’s instructions. The quality of the sequencing library was verified using an Agilent 2200 TapeStation (Agilent). Sequencing analysis was performed using NovaSeq 6000, NovaSeq 6000 S4 reagent kit, and NovaSeq Xp 4-Lane Kit (Illumina) following the manufacturer’s instructions. Low-quality reads were removed using Trimmomatic ^2^ version 0.32. Read mapping on a genomic sequence (UCSC mm10) was performed using BWA-MEM^3^ version 0.7.12. Using the mapping results, copy number alterations were detected using Control-FREEC ^4^ version 8.7, with the following parameters: ploidy 2, window 3000, step 1000, sex XY, and smf contaminationAdjusment FALSE. The gemMappabilityFile was created using the GEM library ^5^ version 1.315, and dbSNP build 142 was utilized for SNPfile. The Control-FREEC output was visualized by running the makeGraph function in R ^6^.

**SUPPLEMENTARY REFERENCES**

1. Zhou Y*, et al.* Metascape provides a biologist-oriented resource for the analysis of systems-level datasets. Nat Commun. 2019;10(1):1523.

2. Bolger AM, Lohse M, Usadel B. Trimmomatic: a flexible trimmer for Illumina sequence data. Bioinformatics. 2014;30(15):2114-20.

3. Li H. Aligning sequence reads, clone sequences and assembly contigs with BWA-MEM 2013 [Available from: https://arxiv.org/abs/1303.3997.

4. Boeva V*, et al.* Control-FREEC: a tool for assessing copy number and allelic content using next-generation sequencing data. Bioinformatics. 2012;28(3):423-5.

5. Derrien T*, et al.* Fast computation and applications of genome mappability. PLoS One. 2012;7(1):e30377.

6. Valentina Boeva MDS. makeGraph.R: 2017 [Available from: https://github.com/BoevaLab/FREEC/blob/master/scripts/makeGraph.R.

**SUPPLEMENTARY FIGURE LEGENDS**

**Figure S1. Generation of murine gall bladder (GB) organoids with *Kras^G12D^* and *Trp53* deletion.**

(A) Murine GB organoids. The representative phase-contrast image in the Matrigel is shown. Scale bar, 200 μm. (B) Cre-mediated removal of the Stop codon in the LSL-cassette. Representative results of genomic PCR are shown. The expected bands were 622 bp for the WT allele (*Kras*^WT^), 500 bp for the LSL cassette (*Kras*^LSL-G12D^), and 650 bp for the recombined allele (*Kras*^G12D^). (C) Organoid successfully transfected with the CRISPR-Cas9 vectors. Green fluorescent protein (GFP) was the reporter for transfection. (D) Genome editing of *Trp53* by CRISPR-Cas9. Chromatograms for both gene-edited and unedited *K*org are shown. The sequences highlighted in green and red denote the targets of double nickase CRISPR/Cas9 vectors. (E) Loss of p53 protein. Western blotting revealed the complete loss of p53 in two independently established clones, *K/53*org #1 and #2. (F, G) Functional analysis of *K/53*org #2. **,*significant (*p*< 0.05). (F) Cell proliferation assay. (G) Flow cytometry-based evaluation. Left panel: evaluation of Ki-67; middle and right panels: dead cells and apoptotic cells, respectively.

**Figure S2. Generation of gall bladder (GB) organoids with *Kras^G12D^* and Smad4 loss or p19^Arf^ loss.**

(A) Genome editing of *Smad4* by CRISPR-Cas9. Chromatograms for both gene-edited and unedited *K*org are shown. The sequences highlighted in green or red denote the targets of double nickase CRISPR/Cas9 vectors. (B) Genome editing of *p19^Arf^* by CRISPR-Cas9 in *K*org. (C) Loss of Smad4 at the protein level. Western blotting revealed complete loss of Smad4 in two independent clones, *K/Smad4*org #1 and #2. (D) Loss of p19^Arf^ at the protein level in two independently clones, *K/p19^Arf^*org #1 and #2. (E) Western blotting for p53 in organoids before inoculation. Retention of p53 protein in *K/Smad4*org and *K/p19^Arf^*org is shown. (F) Immunohistochemical staining of p53 in subcutaneous GB cancers. Tumors are derived from three gene-edited *K*org. Typical examples of p53-positive (*closed arrowheads*) and -negative (open arrowheads) cells. Note that, in Trp53 KO tumors, p53 is detected in stromal cells but not in epithelial cells. Scale bar, 500 μm (low magnification), 100 μm (high magnification).

**Figure S3. Low tumorigenic potential of gall bladder (GB) organoids with *Trp53* loss and *Pik3ca^H1047R^*.**

(A) Lentiviral *Cre*-mediated recombination in the two loci. GB organoids from *Rosa26-Pik3ca^H1047R^* and *Trp53^flox/flox^* were lentivirally transduced and genotyped by genomic PCR. Emergence of amplicon indicates successful recombination. pLKO.1 is a backbone empty vector for LV-Cre. (B) Immunoblotting of transduced organoids. Akt activation by phosphorylation at S473 and loss of p53 were confirmed. β-actin serves as a loading control. (C) Morphology of transduced organoids. Representative images by phase-contrast microscopy are shown. Scale bar, 200 μm. (D) Subcutaneous nodules derived from injected transduced organoids. Results from organoids independently generated in two experiments (Ex. 1 and 2) are shown. Scale bar, 10 mm. E. Sliced subcutaneous nodules. Upper panel displays the nodule from Ex. 2 after formalin fixation. Lower panel displays the results of H&E staining of thin-sliced sections. Note that the nodule is basically a cyst and its wall only partially contained an elevated lesion (asterisk). Scale bar, 10 mm. F. H&E staining of thin sections of subcutaneous nodules. Cyst wall consisted of tall columnar cells (Ex. 1) or papillary lesions (Ex. 2, asterisk in Fig. S1E). Scale bar, 50 μm.

**Figure S4. Failure of development of the orthotopic transplant gall bladder (GB) cancer model.**

(A) The GB *in situ*. Note that the GB is normally attached to the abdominal wall via connective tissue (CT). (B) The direct injection of *K/53*org into the GB. (C) The neck of the GB was tied with a thread. (D) The TB was immediately placed inside the lumen of the GB. (E) Failure of tumor development. Note that implanted TB (asterisk) are not attached to the GB wall. Scale bar, 500 μm. DB, debris. (F) Immunohistochemical staining of orthotopic GB cancer. Scale bar, 500 μm. (G) Immunofluorescence staining of orthotopic GB cancer. Proliferating cells (red, Ki-67) and epithelial cells (green, CK19) are stained. The nucleus is visualized by DAPI (blue). Scale bar, 500 μm (low magnification), 100 μm (high magnification).

**Figure S5. Transcriptome of mouse orthotopic gall bladder (GB) cancer.**

(A) Volcano plot of RNA-seq data. Genes that were differentially more than 2-fold between orthotopic GB cancer tumors and normal GB tissues are shown. The red and green dots indicate genes that were up- and down-regulated in the GB cancer group, respectively. (B) Heat map data generated by setting FDR cut-off at 0.01. Scaling of data was performed by row (gene). GBC, GB cancer group. GB, normal GB group.

**Figure S6. Comparison of transcriptome between mouse and human gall bladder (GB) cancer.**

(A) Network layout of pathway enrichment analysis. Human GB cancer gene expression data were used. Top 20 statistically enriched terms are shown with their *p*-values. Log10(P), the p-value in log base 10. (B) Correlation analysis of DEGs in human and mouse GB cancer tissues. The 532 genes that commonly vary between humans and mice data sets were plotted. Pearson's correlation analysis was performed to analyze the correlation between them (r = 0.33, *p* < 0.0001). GBC, GB cancer group. GB, normal GB group.

**Figure S7. The gating strategy for flow cytometric analysis of immune cells.**

Schematic representation of gating strategy for flow cytometry. CD3ε, CD19, and NK1.1 refer to the dump channel to exclude lineage cells.
