# Supplementary material for: Precision modeling of gall bladder cancer patients in mice based on orthotopic implantation of organoid-derived tumor buds
Source: Oncogenesis. 2021 Apr 17;10(4):33. doi: 10.1038/s41389-021-00322-1 (PMC8053198; doi:10.1038/s41389-021-00322-1)
Supplement: Supplementary file 2 — Supplementary Figures and Tables [file 41389_2021_322_MOESM2_ESM.pptx]

## Slide 1
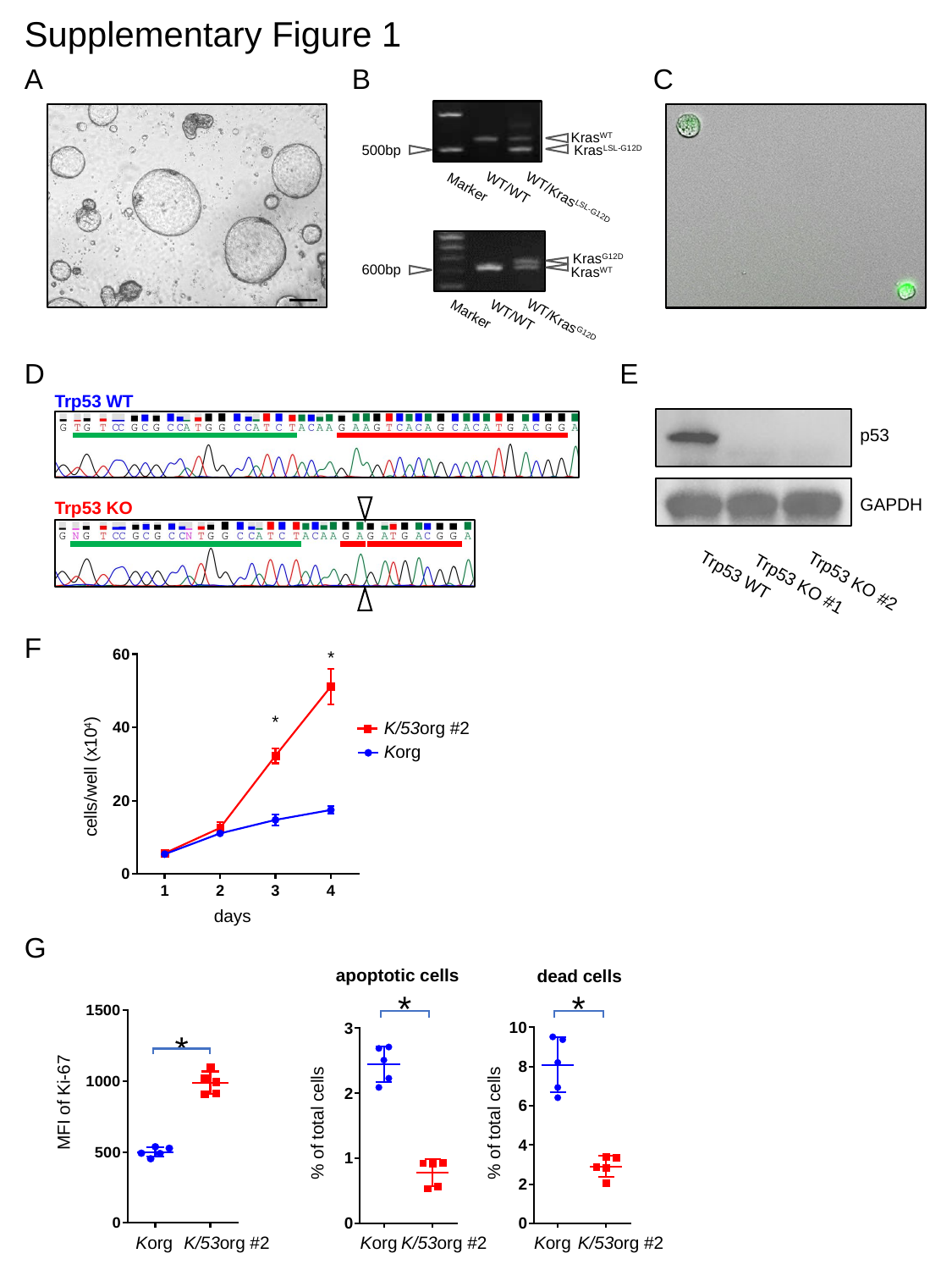

Supplementary Figure 1
A
B
C
KrasWT
500bp
KrasLSL-G12D
WT/WT
Marker
WT/KrasLSL-G12D
KrasG12D
600bp
KrasWT
WT/WT
Marker
WT/KrasG12D
D
E
Trp53 WT
p53
GAPDH
Trp53 KO
Trp53 WT
Trp53 KO #2
Trp53 KO #1
F
K/53org #2
cells/well (x104)
Korg
days
G
apoptotic cells
dead cells
*
*
% of total cells
% of total cells
Korg
K/53org #2
Korg
K/53org #2
*
MFI of Ki-67
Korg
K/53org #2

## Slide 2
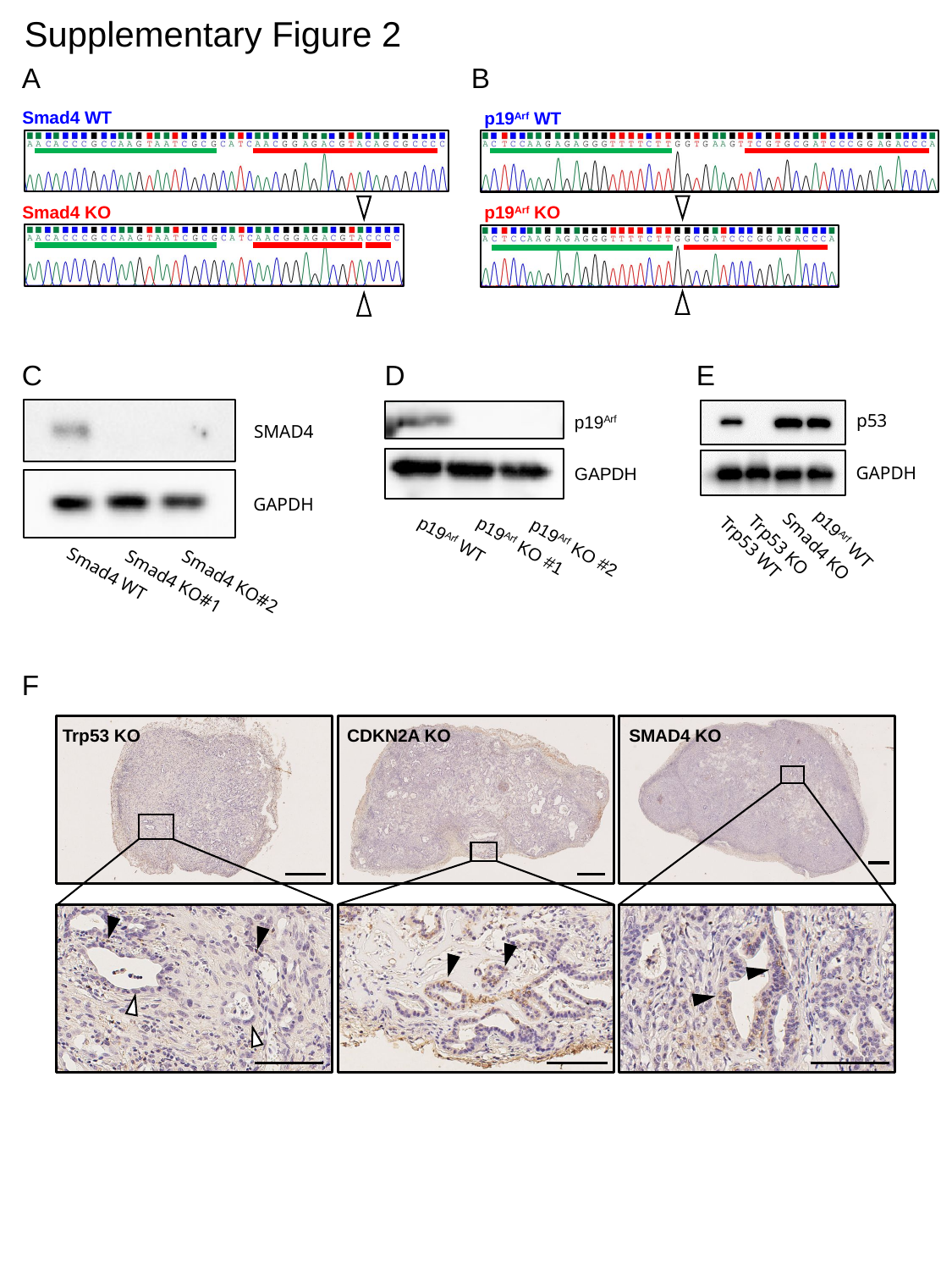

Supplementary Figure 2
A
B
Smad4 WT
Smad4 KO
p19Arf WT
p19Arf KO
C
D
E
SMAD4
GAPDH
Smad4 KO#1
Smad4 KO#2
Smad4 WT
p53
GAPDH
p19Arf WT
Smad4 KO
Trp53 KO
Trp53 WT
p19Arf
GAPDH
p19Arf KO #1
p19Arf KO #2
p19Arf WT
F
Trp53 KO
CDKN2A KO
SMAD4 KO

## Slide 3
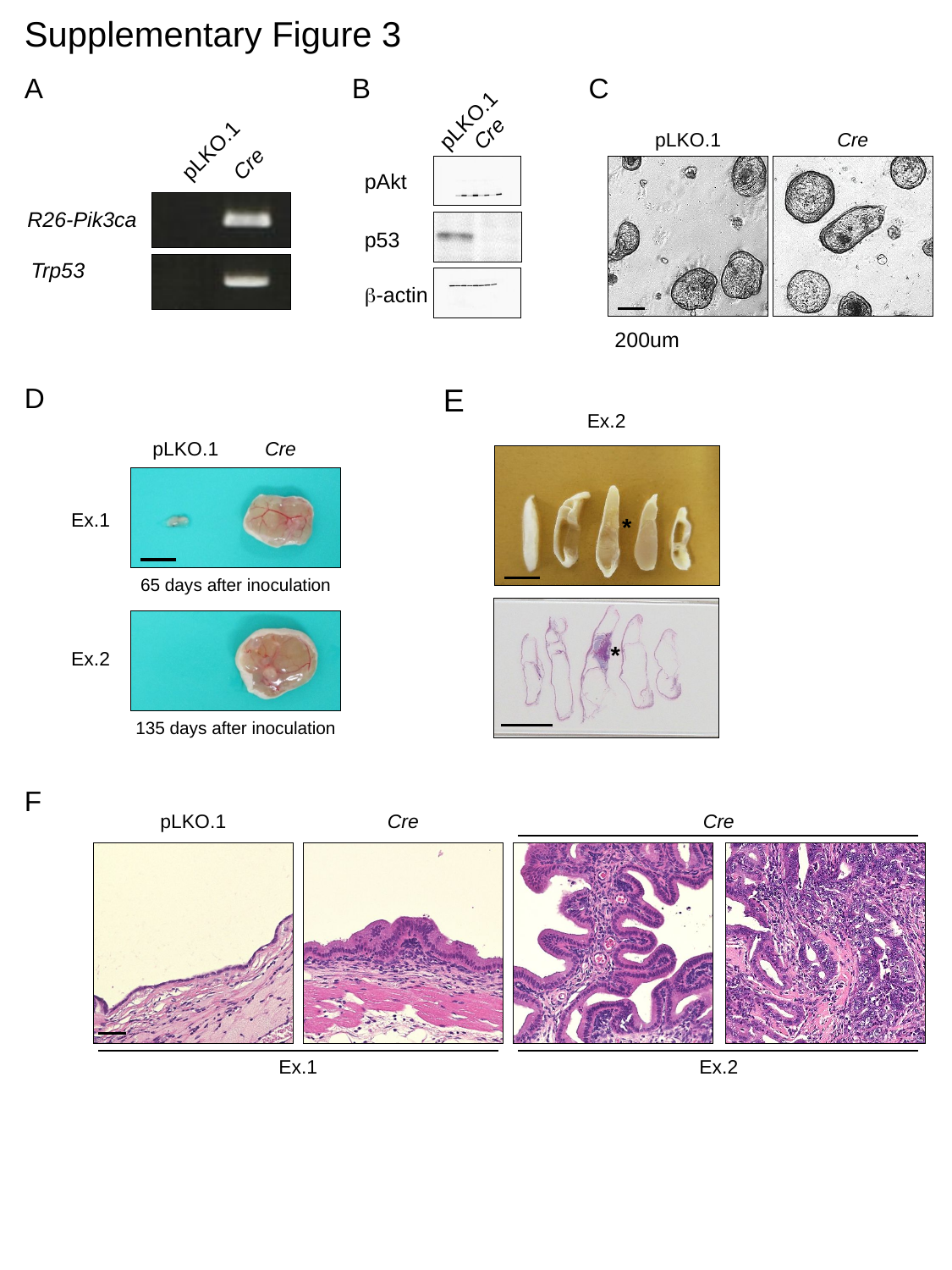

Supplementary Figure 3
A
B
C
pLKO.1
Cre
pAkt
p53
b-actin
pLKO.1
Cre
pLKO.1
Cre
R26-Pik3ca
Trp53
200um
D
E
Ex.2
*
*
pLKO.1
Cre
Ex.1
65 days after inoculation
Ex.2
135 days after inoculation
F
pLKO.1
Cre
Cre
Ex.1
Ex.2

## Slide 4
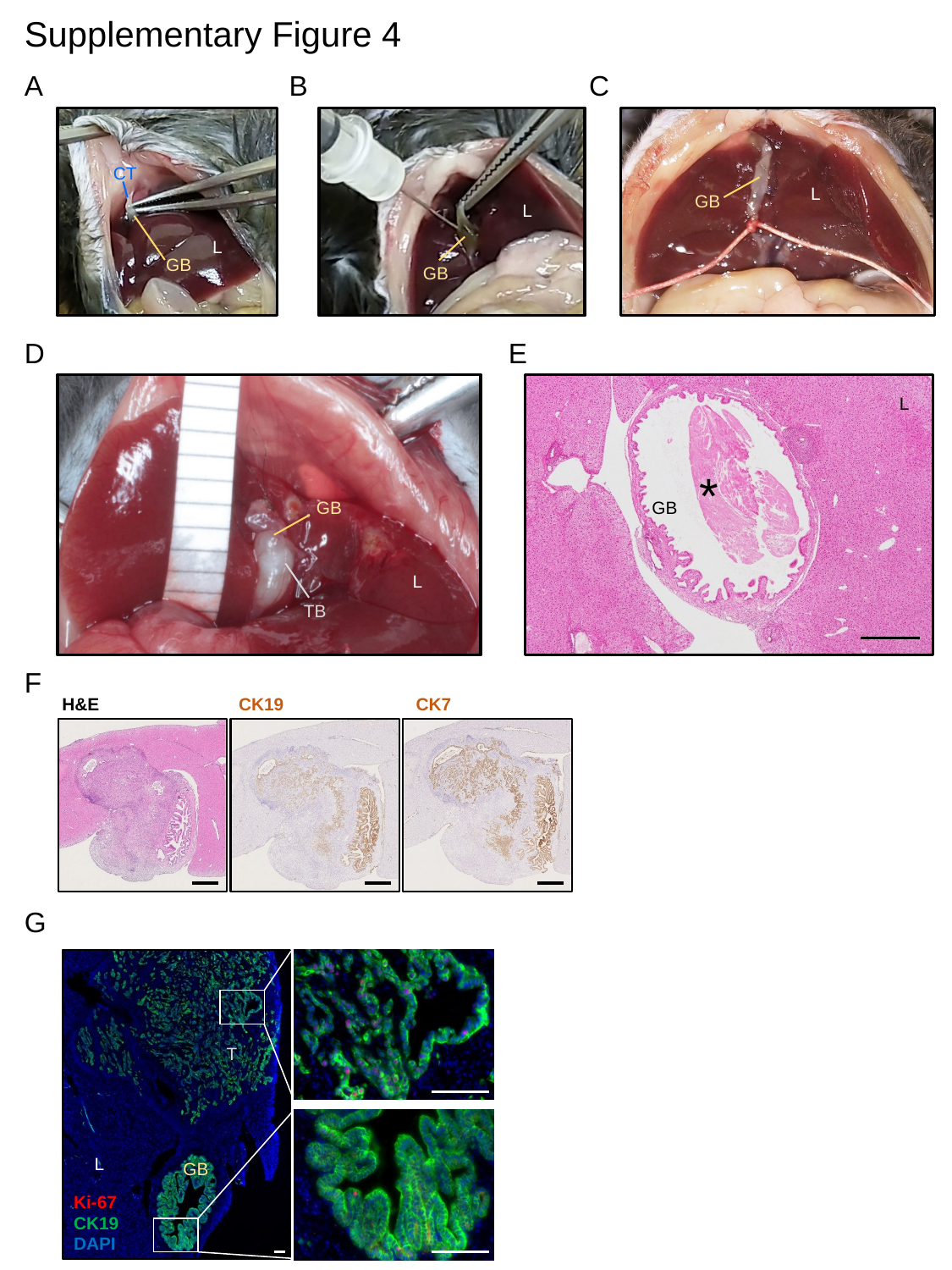

Supplementary Figure 4
A
B
C
CT
L
GB
L
L
GB
GB
D
E
L
*
GB
GB
L
TB
F
H&E
CK19
CK7
G
T
L
GB
Ki-67
CK19
DAPI

## Slide 5
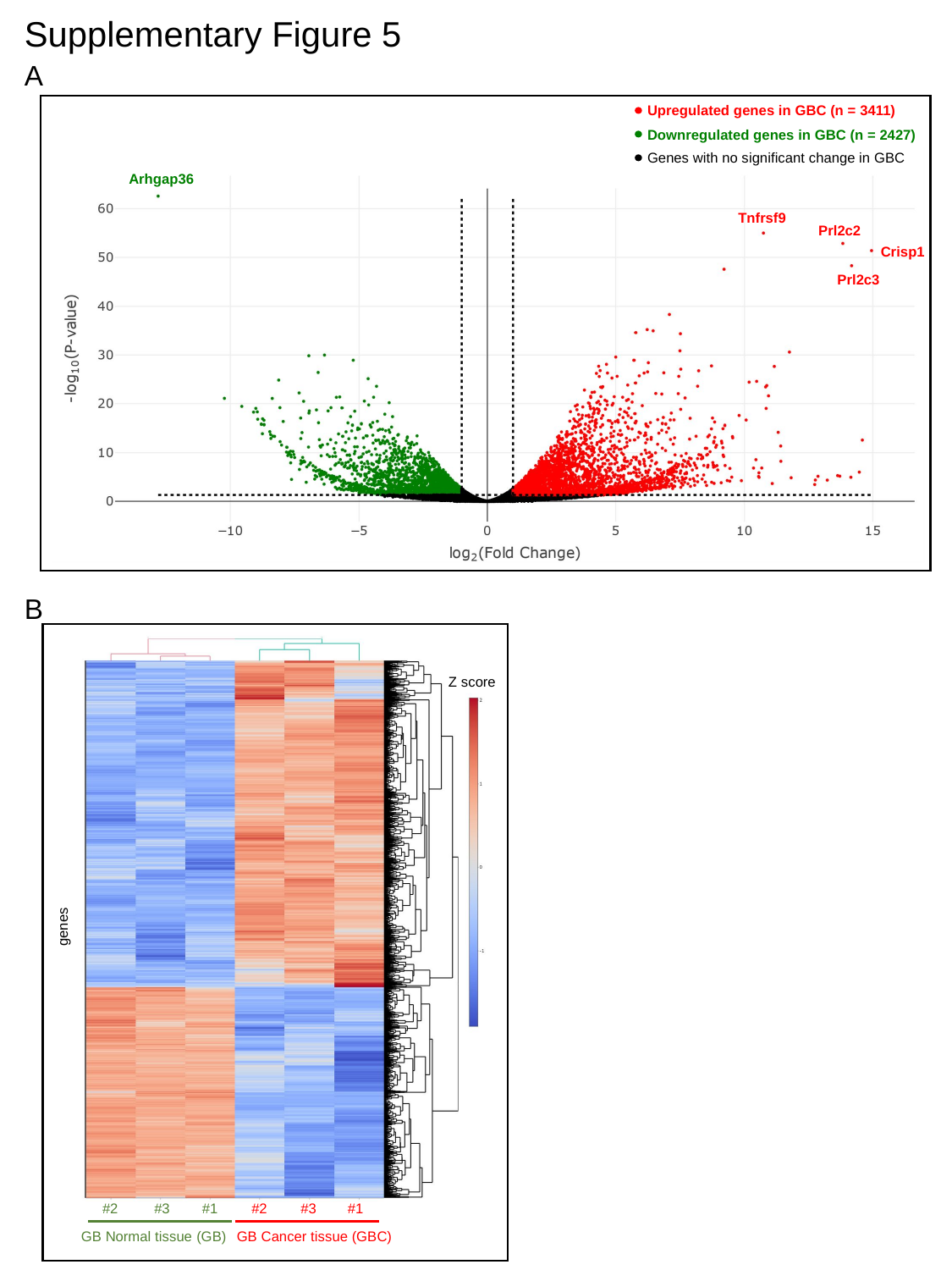

Supplementary Figure 5
A
Upregulated genes in GBC (n = 3411)
Downregulated genes in GBC (n = 2427)
Genes with no significant change in GBC
Arhgap36
Tnfrsf9
Prl2c2
Crisp1
Prl2c3
Log2 (Fold Change)
B
Z score
genes
#2
#3
#1
#2
#3
#1
GB Normal tissue (GB)
GB Cancer tissue (GBC)

## Slide 6
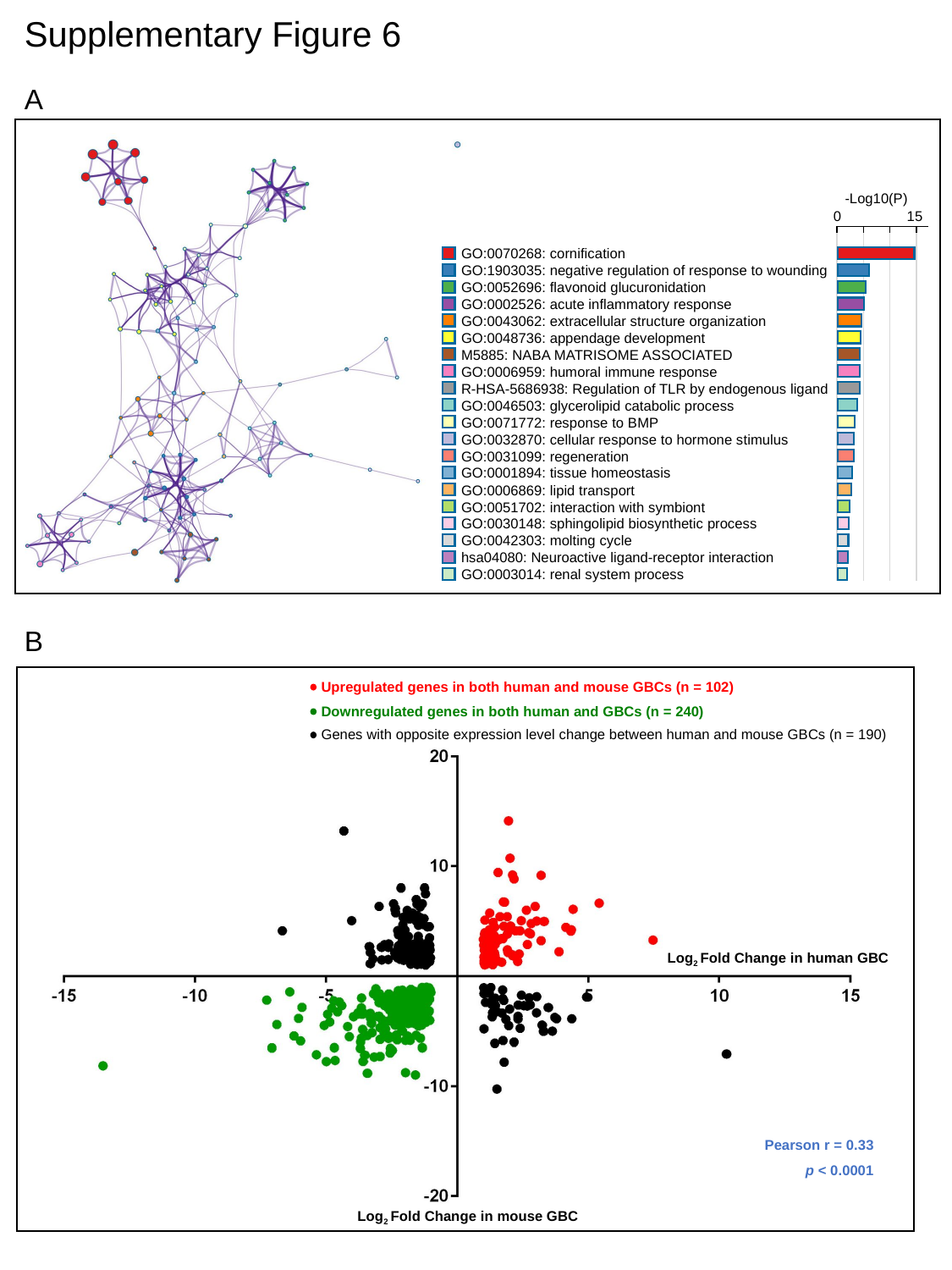

Supplementary Figure 6
A
-Log10(P)
0
15
GO:0070268: cornification
GO:1903035: negative regulation of response to wounding
GO:0052696: flavonoid glucuronidation
GO:0002526: acute inflammatory response
GO:0043062: extracellular structure organization
GO:0048736: appendage development
M5885: NABA MATRISOME ASSOCIATED
GO:0006959: humoral immune response
R-HSA-5686938: Regulation of TLR by endogenous ligand
GO:0046503: glycerolipid catabolic process
GO:0071772: response to BMP
GO:0032870: cellular response to hormone stimulus
GO:0031099: regeneration
GO:0001894: tissue homeostasis
GO:0006869: lipid transport
GO:0051702: interaction with symbiont
GO:0030148: sphingolipid biosynthetic process
GO:0042303: molting cycle
hsa04080: Neuroactive ligand-receptor interaction
GO:0003014: renal system process
B
Upregulated genes in both human and mouse GBCs (n = 102)
Downregulated genes in both human and GBCs (n = 240)
Genes with opposite expression level change between human and mouse GBCs (n = 190)
Log2 Fold Change in human GBC
Pearson r = 0.33
p < 0.0001
Log2 Fold Change in mouse GBC

## Slide 7
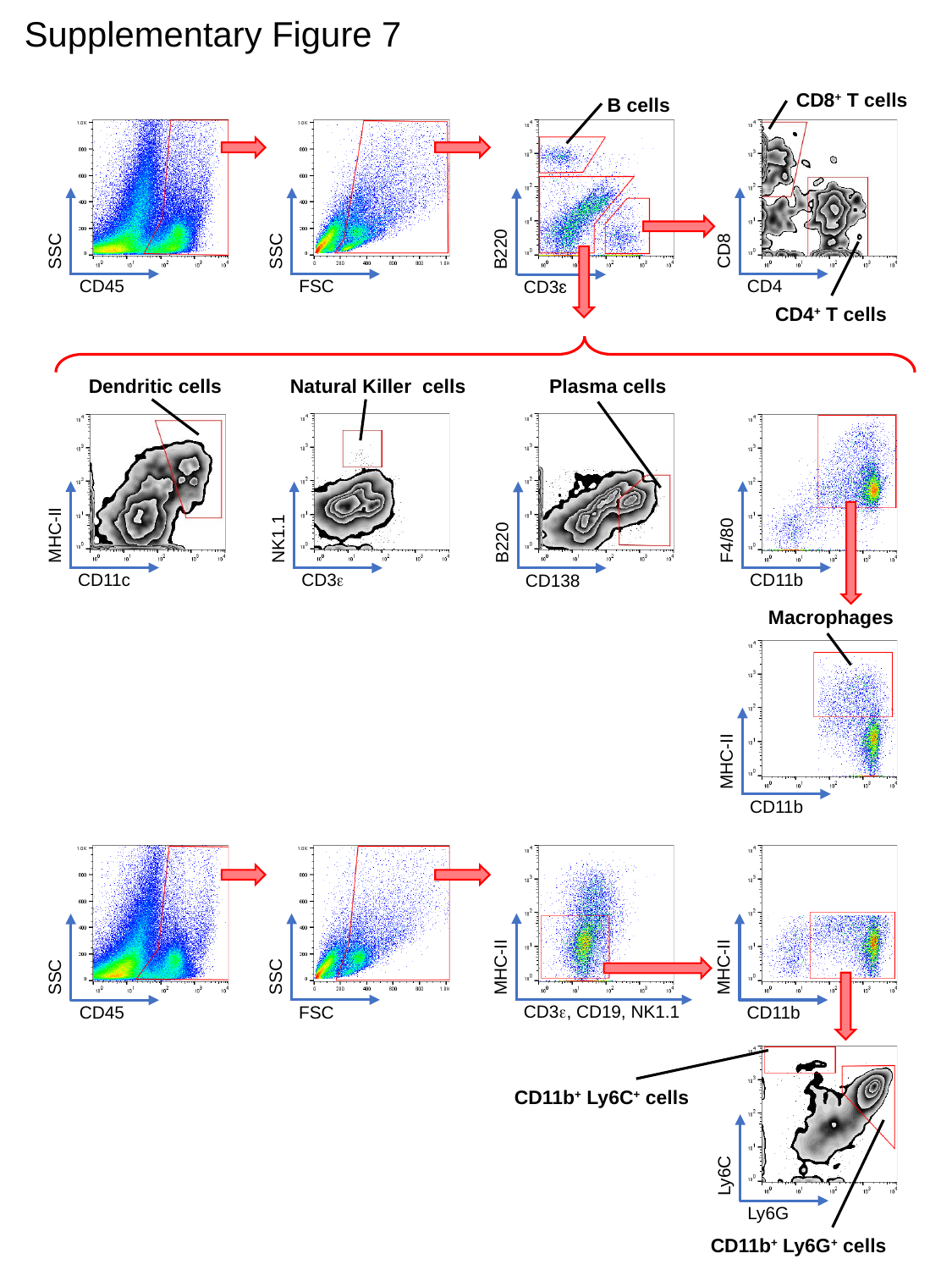

Supplementary Figure 7
CD8+ T cells
B cells
SSC
CD45
SSC
FSC
B220
CD3ɛ
CD8
CD4
CD4+ T cells
Dendritic cells
 Natural Killer cells
 Plasma cells
B220
CD138
NK1.1
CD3e
MHC-II
CD11c
F4/80
CD11b
 Macrophages
MHC-II
CD11b
SSC
CD45
SSC
FSC
MHC-II
CD3e, CD19, NK1.1
MHC-II
CD11b
Ly6C
Ly6G
 CD11b+ Ly6C+ cells
 CD11b+ Ly6G+ cells

## Slide 8
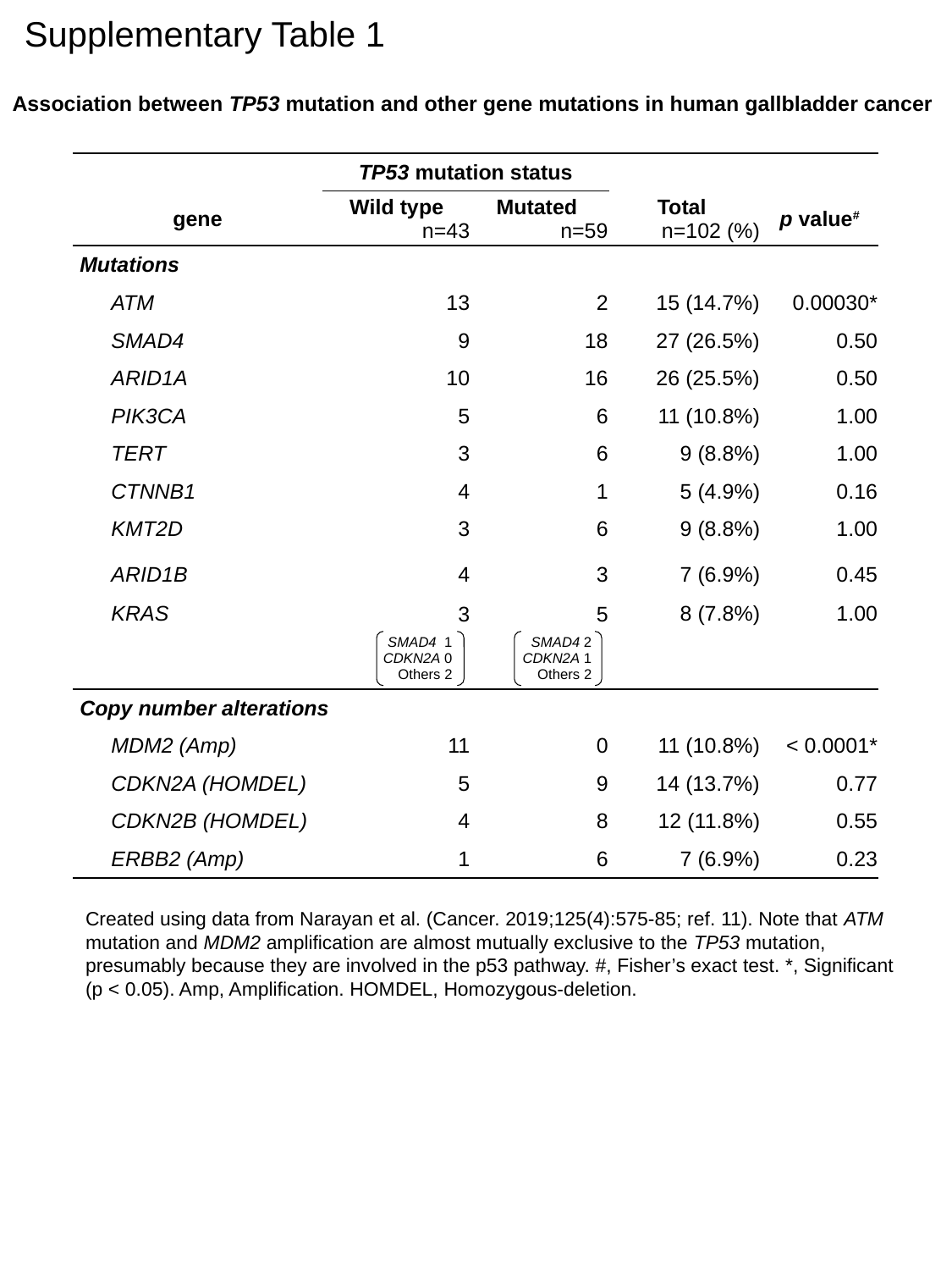

Supplementary Table 1
Association between TP53 mutation and other gene mutations in human gallbladder cancer
| | | TP53 mutation status | | | | | |
| --- | --- | --- | --- | --- | --- | --- | --- |
| gene | | Wild type n=43 | | Mutated n=59 | | Total n=102 (%) | p value# |
| Mutations | | | | | | | |
| | ATM | 13 | | 2 | | 15 (14.7%) | 0.00030\* |
| | SMAD4 | 9 | | 18 | | 27 (26.5%) | 0.50 |
| | ARID1A | 10 | | 16 | | 26 (25.5%) | 0.50 |
| | PIK3CA | 5 | | 6 | | 11 (10.8%) | 1.00 |
| | TERT | 3 | | 6 | | 9 (8.8%) | 1.00 |
| | CTNNB1 | 4 | | 1 | | 5 (4.9%) | 0.16 |
| | KMT2D | 3 | | 6 | | 9 (8.8%) | 1.00 |
| | ARID1B | 4 | | 3 | | 7 (6.9%) | 0.45 |
| | KRAS | | 3 | | 5 | 8 (7.8%) | 1.00 |
| | | SMAD4 1 CDKN2A 0 Others 2 | | SMAD4 2 CDKN2A 1 Others 2 | | | |
| Copy number alterations | | | | | | | |
| | MDM2 (Amp) | 11 | | 0 | | 11 (10.8%) | < 0.0001\* |
| | CDKN2A (HOMDEL) | 5 | | 9 | | 14 (13.7%) | 0.77 |
| | CDKN2B (HOMDEL) | 4 | | 8 | | 12 (11.8%) | 0.55 |
| | ERBB2 (Amp) | 1 | | 6 | | 7 (6.9%) | 0.23 |
| |
| --- |
| |
| --- |
Created using data from Narayan et al. (Cancer. 2019;125(4):575-85; ref. 11). Note that ATM mutation and MDM2 amplification are almost mutually exclusive to the TP53 mutation, presumably because they are involved in the p53 pathway. #, Fisher’s exact test. *, Significant (p < 0.05). Amp, Amplification. HOMDEL, Homozygous-deletion.

## Slide 9
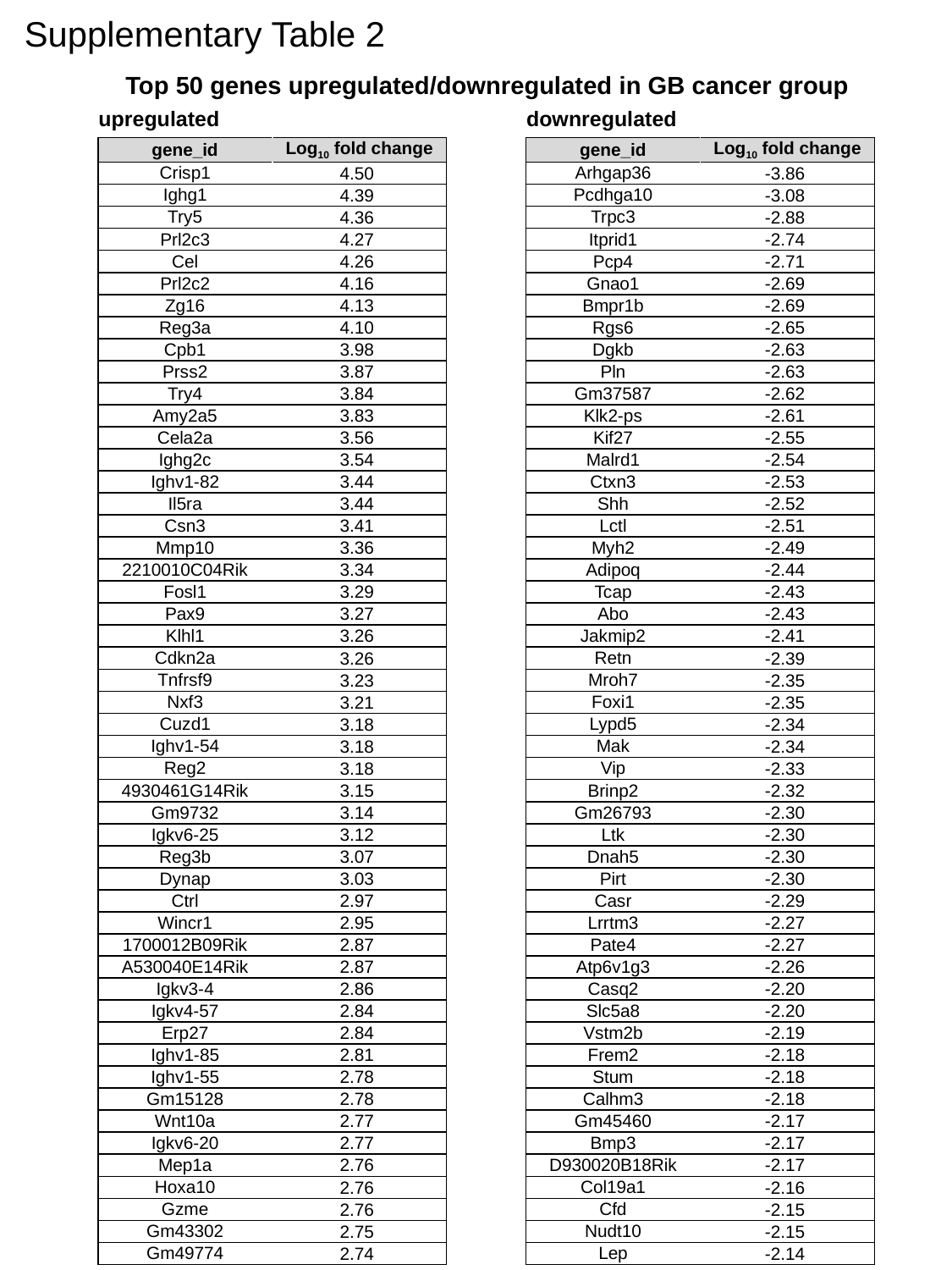

Supplementary Table 2
Top 50 genes upregulated/downregulated in GB cancer group
upregulated
downregulated
| gene\_id | Log10 fold change |
| --- | --- |
| Crisp1 | 4.50 |
| Ighg1 | 4.39 |
| Try5 | 4.36 |
| Prl2c3 | 4.27 |
| Cel | 4.26 |
| Prl2c2 | 4.16 |
| Zg16 | 4.13 |
| Reg3a | 4.10 |
| Cpb1 | 3.98 |
| Prss2 | 3.87 |
| Try4 | 3.84 |
| Amy2a5 | 3.83 |
| Cela2a | 3.56 |
| Ighg2c | 3.54 |
| Ighv1-82 | 3.44 |
| Il5ra | 3.44 |
| Csn3 | 3.41 |
| Mmp10 | 3.36 |
| 2210010C04Rik | 3.34 |
| Fosl1 | 3.29 |
| Pax9 | 3.27 |
| Klhl1 | 3.26 |
| Cdkn2a | 3.26 |
| Tnfrsf9 | 3.23 |
| Nxf3 | 3.21 |
| Cuzd1 | 3.18 |
| Ighv1-54 | 3.18 |
| Reg2 | 3.18 |
| 4930461G14Rik | 3.15 |
| Gm9732 | 3.14 |
| Igkv6-25 | 3.12 |
| Reg3b | 3.07 |
| Dynap | 3.03 |
| Ctrl | 2.97 |
| Wincr1 | 2.95 |
| 1700012B09Rik | 2.87 |
| A530040E14Rik | 2.87 |
| Igkv3-4 | 2.86 |
| Igkv4-57 | 2.84 |
| Erp27 | 2.84 |
| Ighv1-85 | 2.81 |
| Ighv1-55 | 2.78 |
| Gm15128 | 2.78 |
| Wnt10a | 2.77 |
| Igkv6-20 | 2.77 |
| Mep1a | 2.76 |
| Hoxa10 | 2.76 |
| Gzme | 2.76 |
| Gm43302 | 2.75 |
| Gm49774 | 2.74 |
| gene\_id | Log10 fold change |
| --- | --- |
| Arhgap36 | -3.86 |
| Pcdhga10 | -3.08 |
| Trpc3 | -2.88 |
| Itprid1 | -2.74 |
| Pcp4 | -2.71 |
| Gnao1 | -2.69 |
| Bmpr1b | -2.69 |
| Rgs6 | -2.65 |
| Dgkb | -2.63 |
| Pln | -2.63 |
| Gm37587 | -2.62 |
| Klk2-ps | -2.61 |
| Kif27 | -2.55 |
| Malrd1 | -2.54 |
| Ctxn3 | -2.53 |
| Shh | -2.52 |
| Lctl | -2.51 |
| Myh2 | -2.49 |
| Adipoq | -2.44 |
| Tcap | -2.43 |
| Abo | -2.43 |
| Jakmip2 | -2.41 |
| Retn | -2.39 |
| Mroh7 | -2.35 |
| Foxi1 | -2.35 |
| Lypd5 | -2.34 |
| Mak | -2.34 |
| Vip | -2.33 |
| Brinp2 | -2.32 |
| Gm26793 | -2.30 |
| Ltk | -2.30 |
| Dnah5 | -2.30 |
| Pirt | -2.30 |
| Casr | -2.29 |
| Lrrtm3 | -2.27 |
| Pate4 | -2.27 |
| Atp6v1g3 | -2.26 |
| Casq2 | -2.20 |
| Slc5a8 | -2.20 |
| Vstm2b | -2.19 |
| Frem2 | -2.18 |
| Stum | -2.18 |
| Calhm3 | -2.18 |
| Gm45460 | -2.17 |
| Bmp3 | -2.17 |
| D930020B18Rik | -2.17 |
| Col19a1 | -2.16 |
| Cfd | -2.15 |
| Nudt10 | -2.15 |
| Lep | -2.14 |

## Slide 10
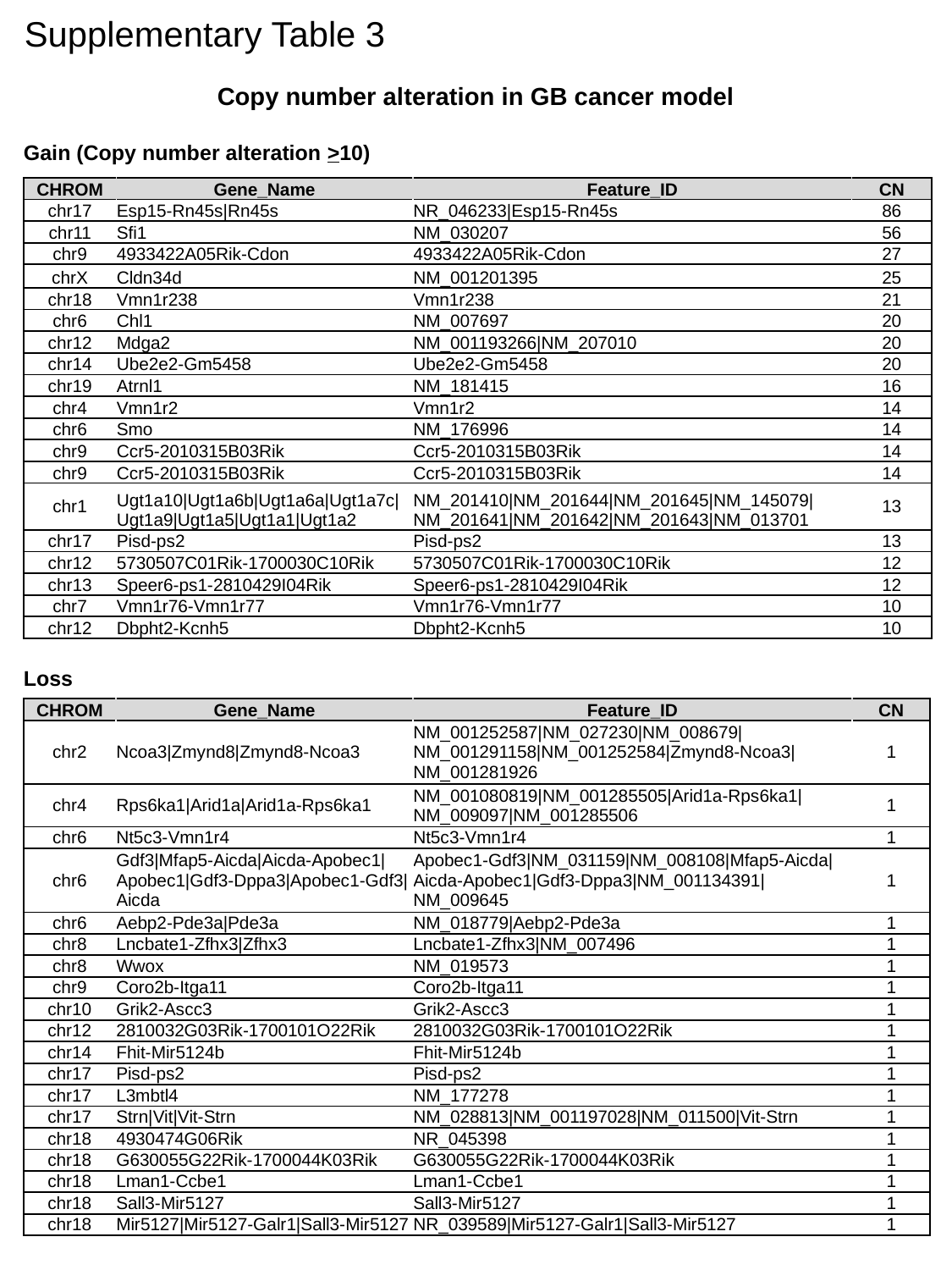

Supplementary Table 3
Copy number alteration in GB cancer model
Gain (Copy number alteration >10)
| CHROM | Gene\_Name | Feature\_ID | CN |
| --- | --- | --- | --- |
| chr17 | Esp15-Rn45s|Rn45s | NR\_046233|Esp15-Rn45s | 86 |
| chr11 | Sfi1 | NM\_030207 | 56 |
| chr9 | 4933422A05Rik-Cdon | 4933422A05Rik-Cdon | 27 |
| chrX | Cldn34d | NM\_001201395 | 25 |
| chr18 | Vmn1r238 | Vmn1r238 | 21 |
| chr6 | Chl1 | NM\_007697 | 20 |
| chr12 | Mdga2 | NM\_001193266|NM\_207010 | 20 |
| chr14 | Ube2e2-Gm5458 | Ube2e2-Gm5458 | 20 |
| chr19 | Atrnl1 | NM\_181415 | 16 |
| chr4 | Vmn1r2 | Vmn1r2 | 14 |
| chr6 | Smo | NM\_176996 | 14 |
| chr9 | Ccr5-2010315B03Rik | Ccr5-2010315B03Rik | 14 |
| chr9 | Ccr5-2010315B03Rik | Ccr5-2010315B03Rik | 14 |
| chr1 | Ugt1a10|Ugt1a6b|Ugt1a6a|Ugt1a7c|Ugt1a9|Ugt1a5|Ugt1a1|Ugt1a2 | NM\_201410|NM\_201644|NM\_201645|NM\_145079|NM\_201641|NM\_201642|NM\_201643|NM\_013701 | 13 |
| chr17 | Pisd-ps2 | Pisd-ps2 | 13 |
| chr12 | 5730507C01Rik-1700030C10Rik | 5730507C01Rik-1700030C10Rik | 12 |
| chr13 | Speer6-ps1-2810429I04Rik | Speer6-ps1-2810429I04Rik | 12 |
| chr7 | Vmn1r76-Vmn1r77 | Vmn1r76-Vmn1r77 | 10 |
| chr12 | Dbpht2-Kcnh5 | Dbpht2-Kcnh5 | 10 |
Loss
| CHROM | Gene\_Name | Feature\_ID | CN |
| --- | --- | --- | --- |
| chr2 | Ncoa3|Zmynd8|Zmynd8-Ncoa3 | NM\_001252587|NM\_027230|NM\_008679|NM\_001291158|NM\_001252584|Zmynd8-Ncoa3|NM\_001281926 | 1 |
| chr4 | Rps6ka1|Arid1a|Arid1a-Rps6ka1 | NM\_001080819|NM\_001285505|Arid1a-Rps6ka1|NM\_009097|NM\_001285506 | 1 |
| chr6 | Nt5c3-Vmn1r4 | Nt5c3-Vmn1r4 | 1 |
| chr6 | Gdf3|Mfap5-Aicda|Aicda-Apobec1|Apobec1|Gdf3-Dppa3|Apobec1-Gdf3|Aicda | Apobec1-Gdf3|NM\_031159|NM\_008108|Mfap5-Aicda|Aicda-Apobec1|Gdf3-Dppa3|NM\_001134391|NM\_009645 | 1 |
| chr6 | Aebp2-Pde3a|Pde3a | NM\_018779|Aebp2-Pde3a | 1 |
| chr8 | Lncbate1-Zfhx3|Zfhx3 | Lncbate1-Zfhx3|NM\_007496 | 1 |
| chr8 | Wwox | NM\_019573 | 1 |
| chr9 | Coro2b-Itga11 | Coro2b-Itga11 | 1 |
| chr10 | Grik2-Ascc3 | Grik2-Ascc3 | 1 |
| chr12 | 2810032G03Rik-1700101O22Rik | 2810032G03Rik-1700101O22Rik | 1 |
| chr14 | Fhit-Mir5124b | Fhit-Mir5124b | 1 |
| chr17 | Pisd-ps2 | Pisd-ps2 | 1 |
| chr17 | L3mbtl4 | NM\_177278 | 1 |
| chr17 | Strn|Vit|Vit-Strn | NM\_028813|NM\_001197028|NM\_011500|Vit-Strn | 1 |
| chr18 | 4930474G06Rik | NR\_045398 | 1 |
| chr18 | G630055G22Rik-1700044K03Rik | G630055G22Rik-1700044K03Rik | 1 |
| chr18 | Lman1-Ccbe1 | Lman1-Ccbe1 | 1 |
| chr18 | Sall3-Mir5127 | Sall3-Mir5127 | 1 |
| chr18 | Mir5127|Mir5127-Galr1|Sall3-Mir5127 | NR\_039589|Mir5127-Galr1|Sall3-Mir5127 | 1 |
